# Automating Model Building for SPM Images of Biomolecules Using MISO

**DOI:** 10.1021/acs.jcim.6c01134

**Published:** 2026-08-03

**Authors:** Claudia L. Gomez-Flores, Kelvin Anggara

**Affiliations:** 28326 Max Planck Institute for Solid State Research, Heisenbergstrasse 1, Stuttgart 70569, Germany

## Abstract

Direct imaging by scanning probe microscopy (SPM) at cryogenic temperatures in ultrahigh vacuum (UHV) has enabled structural characterization of individual biomolecules in heterogeneous systems, including glycans and glycan-decorated biomolecules (also known as glycoconjugates). However, interpreting SPM images into molecular structures remains a major bottleneck, particularly for flexible biomolecules that can adopt multiple adsorption geometries. Currently, hypothesizing, constructing, and testing these geometries are largely performed manually, thus limiting the data throughput of SPM-based structural analysis and the scale of molecular systems addressable by SPM imaging technologies. Here, we present MISO (Model building from Identity, Sequence, and Observed location) as a workflow for generating three-dimensional biomolecular models from user-defined hypotheses about subunit identity, connectivity, and observed locations in SPM images. MISO produces structures consistent with user-defined hypotheses by implementing the quaternion estimator algorithm (QUEST) followed by biased classical molecular dynamics (MD). When applied to existing SPM data of glycans and glycoconjugates, MISO generated structures that were qualitatively consistent with structures previously validated by density functional theory (DFT) calculations. In addition, we show the use of MISO in filtering user hypotheses as well as the scale-up potential of MISO in building a 3D model of an unfolded glycoprotein on the surface. These results establish MISO as a practical tool for automating molecular model building, which helps to improve the overall throughput of SPM image interpretation of flexible biomolecules.

## Graphical abstract

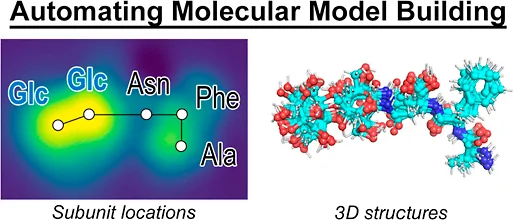

Direct imaging of single molecules is one of the simplest means to determine their individual structures; a feat that has so far only been demonstrated by scanning probe microscopy (SPM) of molecules on surfaces. Direct SPM imaging offers nondestructive visualization of individual molecules at near-atomic resolution that reveals how their subunits or even atoms connect to one another.^1–3^ As a proof-of-concept, direct SPM imaging at cryogenic temperatures in ultrahigh vacuum (UHV) has enabled first-time nanoscale characterization of previously unknown molecular systems, including natural products,^3^ extraterrestrial organic materials,^4,5^ combustion products,^6^ conjugated polymers,^7,8^ and biomolecules.^9^

In the context of biomolecules, as a recent example, direct SPM imaging demonstrates promising steps toward single-molecule analysis of glycans (also known as carbohydrates or oligosaccharide), which are commonly found in biological systems attached to lipids, proteins, or RNAs as glycolipids, glycoproteins, or glycoRNAs^10–12^ (also known as glycoconjugates). SPM imaging at cryogenic temperatures provides direct access to individual structures of glycans^13–16^ and glyconjugates,^9^ revealing how every monosaccharide subunit connects to other monosaccharides, amino acid, or lipid moieties in biologically sourced glycopeptides, glycolipids, and glycoproteins. In doing so, direct SPM imaging shows its potential in analyzing structures of biological glycans and glycoconjugates, whose high structural diversity poses challenges for today’s ensemble-averaged analytical techniques.^17^ Single-molecule approaches are capable of meeting this challenge, such as direct SPM imaging,^9,13–16^ optical microscopy,^18^ and nanopores,^19–22^ are all being actively developed into high-throughput analytical platforms capable of analyzing structurally diverse glycans and glycoconjugates that arise from the large variety of monosaccharides (so far ∼30 in mammals and ∼500 in bacteria^12,23^ ) and the multiple ways they connect to other monosaccharides, as well as to various lipid, protein, or RNA structures.

However, the prospect of direct SPM imaging as an analytical tool for glycoscience and beyond is hindered by the challenge of its low data throughput, particularly in interpreting/solving SPM images into molecular structures, also known as “model building” in structural biology^24^ or “backmapping” in computational chemistry.^25,26^ The absence of a software environment to generate, manipulate, and fit three-dimensional biomolecular structures into SPM images forces their analysis to be performed entirely manually, which ultimately imposes practical limits to the scale of biomolecular systems addressable by the SPM imaging technology. The software development landscape in the field of low-temperature SPM biomolecular imaging, capable of accessing subunit information^9,13^ and atomic-level geometries^14–16^ of a biomolecule, clearly lags behind the field of ambient SPM biomolecular imaging,^27–32^ which routinely accesses large assemblies of biomolecular structures at solid–liquid interfaces, albeit at lower submicrometer resolutions. Tools developed thus far in low-temperature SPM communities have been highly specialized toward detecting small molecules^33–35^ and interpreting their images into their adsorption geometries.^15,36–39^ As a result, there is no software platform targeted at atomic-level model building of biomolecules, geared toward solving low-temperature SPM images of biomolecules.

A typical workflow for interpreting SPM images of structurally flexible biomolecules involves generating structural hypotheses, building the corresponding molecular models, and simulating SPM images using ab initio methods for comparison with experimental images (Figure 1). The workflow begins with an SPM image of a biomolecule and the relevant chemical information, which may be obtained by mass spectrometry characterization of ions deposited onto the surface and/or by sample preparation methods, such as stereoselective chemical synthesis (e.g., solid-phase synthesis of glycans), enzymatic treatments (e.g., sample treated with ribonuclease to remove RNA), and/or chromatographic purification (e.g., size-exclusion chromatography).

**Figure 1:**
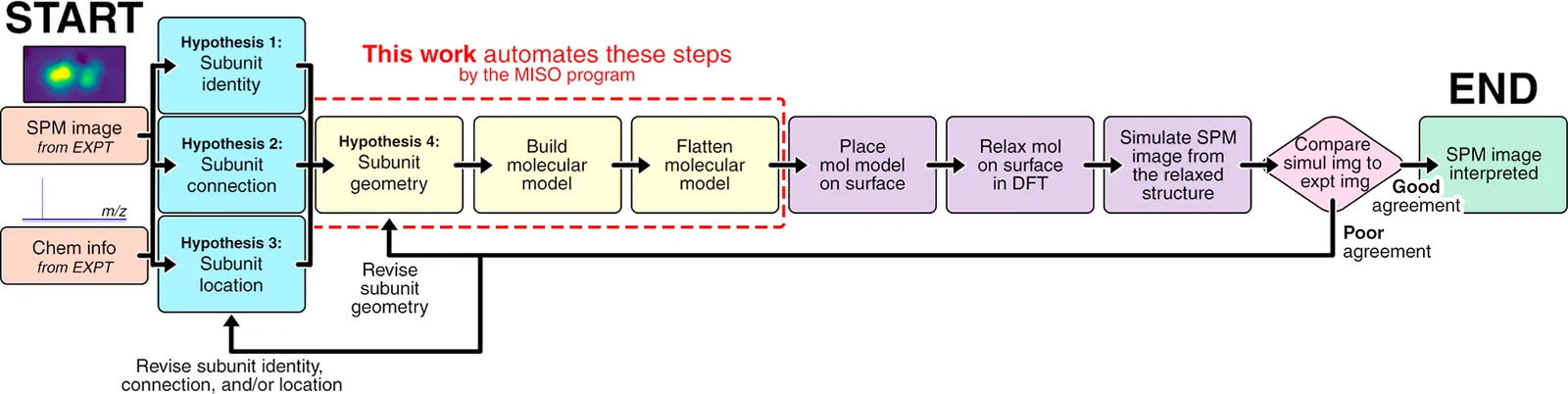
Typical workflow in interpreting an SPM image. Experimental data (e.g., SPM image and chemical information) is used to generate hypotheses on subunit identity, connection, location, and geometry. Molecular models corresponding to these hypotheses are built and flattened to achieve a realistic adsorption geometry. The flattened molecular models are placed on the surface to be relaxed by DFT to give structures from which the SPM image is simulated. If the simulated SPM image agrees with the experimental SPM image, then the SPM image is considered to be successfully interpreted. On the other hand, if the simulated SPM image disagrees with the experimental SPM image, then the structure is rejected and the initial hypotheses must be revised. In this work, we present MISO (Model-building from Identity, Sequence, and Observed-locations) as a tool to automate hypotheses generation concerning subunit geometries as well as building and flattening of the 3D molecular model.

Together, the SPM image and prior chemical information lead to testable structural hypotheses concerning subunit identity, connectivity, location, and geometry (Figure 1). For example, in the case of a glycan, one would hypothesize which monosaccharides are present, how they are connected, where each monosaccharide is located in the SPM image, and whether each pyranose ring lies flat on the surface. Hypotheses about subunit identity and connectivity are often derived from mass spectrometry or constraints imposed during the sample preparation. In contrast, hypotheses about subunit location and geometry are often generated by comparing the SPM image of the biomolecule to reference SPM images of small molecules containing the same functional groups or structural motifs. For example, sulfate groups in glycosaminoglycans are identified by the sulfate feature observed in indoxyl sulfate,^9^ and horizontal glucose subunits in glycoproteins and glycopeptides are identified by the apparent heights of horizontal glucopyranose rings observed in cellohexaose.^41^

To test these hypotheses, 3D molecular models consistent with the proposed subunit identity, connectivity, location, and geometry are manually built using molecular modeling software such as Avogadro.^40^ Building these 3D molecular models from these user hypotheses is an important first filter of the hypotheses—because the 3D model built would immediately reveal any unphysical element that invalidates the hypotheses (e.g., overextended C–C bond, overbent C–O–C glycosidic bond, overlapping atoms, etc.).

These models are then flattened to produce conformations more realistic for adsorption on the surface. Such a flattening procedure is a heuristic to maximize molecule-surface contact that serves to (i) mimic the molecule–surface attraction without explicitly describing surface type and facet and (ii) eliminate unrealistic molecular geometry on the surface (e.g., a benzene ring parallel to the surface that floats above a surface). This flattening step is important because SPM imaging is currently used to study unfolded biomolecules to determine connectivity between their constituent subunits (i.e., their primary structure)—and, often, to obtain a clearer view of how individual subunits are connected. Biomolecules on surfaces are manipulated by the SPM tip to further unfold them, as demonstrated for proteins^9^ and glycans.^41^ Such manipulation converts unstable or poorly imageable features, such as stacked pyranose rings that may be too insulating for STM imaging, into more stable and imageable structures, such as individual pyranose rings lying flat on the surface, which are understood to possess a greater extent of molecule–surface contact.

In the next step of the workflow, each flattened molecular model is transferred onto a surface and relaxed using Density Functional Theory (DFT) to account for quantum mechanical interactions between the molecule and the surface. The resulting relaxed structures are used to simulate SPM images, whereby noncontact AFM images are generated using the probe-particle model,^42^ whereas STM images are simulated from DFT-derived local density of states using the Tersoff–Hamann approximation.^43^ The simulated SPM image for each molecular model is compared with the experimental SPM image: good agreement concludes the SPM image interpretation, whereas poor agreement indicates that the initial hypotheses should be revised. This triggers another cycle of model building, DFT relaxation, image simulation, and image comparison—until a good simulation-experiment agreement is reached. We note that the use of DFT is always required to claim if a molecular structure (whether built manually or by a computer program) interprets an SPM image, as the SPM image simulated from the molecular structure must agree with the experimental SPM image.

In the quest to improve the throughput of SPM image interpretation, we identify two major bottlenecks at the beginning of this workflow that arise from the need for manual work (Figure 1). The first is the need to manually build 3D molecular structures that satisfy the hypothesized subunit identity, connectivity, and location. The second is the need to manually flatten these 3D structures before DFT relaxation on the surface. The first bottleneck arises because molecular model building is typically performed in software that requires users to rotate covalent bonds manually, one bond at a time, in order to explore possible geometries. For example, users may need to rotate the C–O bond of a glycosidic linkage connecting two pyranose rings. This time-consuming process is further exacerbated by the need to position the subunits of the 3D molecular model so that they match the hypothesized subunit locations observed in the 2D SPM image. The second bottleneck is the need to flatten the constructed molecular models onto a 2D plane before DFT relaxation. This step is necessary because DFT relaxation is generally ineffective at bringing elevated or protruding molecular moieties down onto the surface, leading to unrealistic adsorbed structures. This limitation arises because DFT relaxation searches for local potential-energy minima rather than the global minimum.

To address these two bottlenecks, here, we present MISO (Model-building from Identity, Sequence, and Observed-locations) as a tool that generates 3D biomolecular structures from user hypotheses, including subunit identity, connection, and locations. In MISO, hypotheses concerning subunit geometry are automatically generated and systematically assembled into various 3D molecular structures. MISO explores various subunit geometries and assembles them using the quaternion estimator algorithm (QUEST)^44^ to yield multiple 3D molecular models, which are then relaxed using a classical force field modified with biasing forces that flattens the structures on a 2D plane. As the first demonstration, we deploy MISO in the interpretation of SPM images of glycans and glycoconjugates from our previous work^9^ and show that MISO successfully generates 3D structures that are very similar to the manually built 3D structures verified by DFT calculations. We thereby demonstrate MISO as a tool to generate biomolecular structures consistent with experimental observations without manual effort, thus eliminating a key bottleneck in SPM image interpretation work that is notorious for its manual, subjective, expert-driven process. In addition, we also demonstrate the use of MISO in invalidating hypothesized sequences (primary structures) by testing, for every hypothesized sequence, if the corresponding 3D molecular models are physically plausible or not—a feat that would be too time-consuming by manual effort. Finally, we demonstrate the use of MISO in building molecular models for unfolded glycoproteins on the surface, highlighting its scale-up potential to address biomolecular systems typical in biochemical studies in the future. Taken altogether, such capabilities underscore the potential of MISO as a key component that helps advance direct SPM imaging of individual biomolecules as an emerging analytical tool capable of determining individual structures of unknown biomolecules.

## Results and Discussion

### MISO Design

We design MISO to convert user-defined hypotheses of an observed biomolecule into plausible 3D structures (Figure 2). As an input, MISO requires the identity, location, and connectivity of every subunit within the biomolecule, formatted as a text-based file (see examples in the Supporting Information). MISO uses these inputs to generate a 3D structure for each subunit at its specific (*x*, *y*) location before covalently connecting them according to the provided connectivity data. Afterward, the resulting structures are refined by classical force fields to yield the molecular structures, which are ready for subsequent DFT relaxation and SPM image simulation. Comparing the simulated images with the experimental SPM image should reveal which candidate structure is the best solution for interpreting the SPM image.

**Figure 2:**
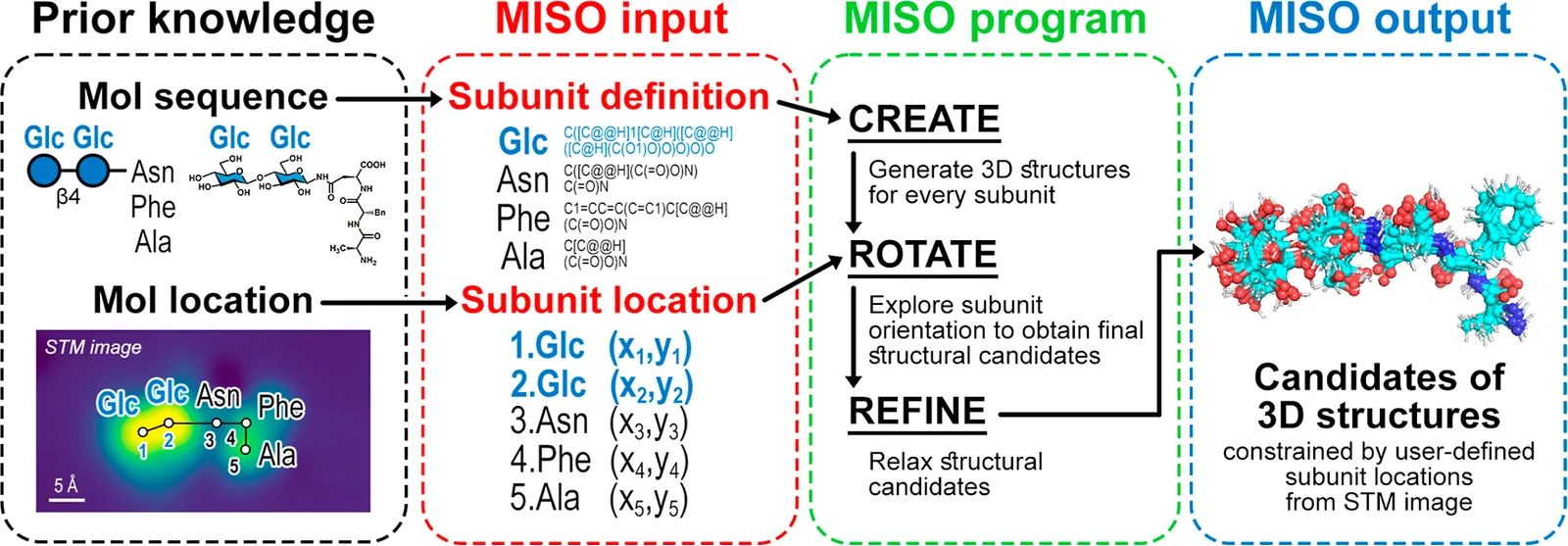
Overview of MISO design. MISO converts user-hypothesized subunit identity, connection, and location within a biomolecule into 3D molecular structures that obey these sequence and location constraints. To accomplish this, MISO uses CREATE, ROTATE, and REFINE pipelines, which are tasked with generating, varying, and relaxing 3D structures of biomolecules (see Methods for details).

In the following, we detail the algorithm used in MISO, which showcases judicious use of applied mathematics in computational chemistry. Figure 2 shows the MISO pipeline to consist of CREATE, where individual subunit structures are generated; ROTATE, where individual subunits are spatially aligned to covalently link them together and to generate multiple structural candidates; and finally REFINE, where structural candidates are relaxed with classical molecular dynamics (MD). Along the MISO pipeline, we especially highlight (i) the use of QUEST in ROTATE to orient every subunit in the molecule and generate multiple 3D structural candidates as well as (ii) the use of classical force field modified with biasing forces in REFINE to access more realistic molecular geometries on the surface. At present, we have deliberately avoided using machine learning (ML) methods due to difficulties in accessing and generating large and reliable training data that are necessary to train useful models. However, we anticipate that the increased number of interpreted/labeled SPM image data generated from continuous use of MISO in real-world SPM laboratories would allow image-to-structure and structure-to-image ML models to be feasible in the near future for SPM biomolecular imaging, given the successes shown by these ML models on SPM imaging of small molecules.^15,33,38,39^

### MISO Input

To generate structures of glycan and glycoconjugates, MISO requires three inputs: (1) a list of monosaccharide subunit identities defined as SMILES (Simplified Molecular Input Line Entry System);^45^ (2) a list of (*x*,*y*) locations of the monosaccharide subunits; and (3) a list of connections between the monosaccharide subunits. For example, to describe a cellobiose, the input file contains SMILES for glucose, the β­(1,4) glycosidic bond between them, as well as the location of the glucose subunit on the (*x*,*y*) plane (see example in Supporting Information).

We note that MISO input requires users to know the full or partial identity of the observed biomolecule. If the sequence (i.e., subunit identity and its connections) and/or the subunit location are only partially known, users would need to run MISO multiple times, each with a unique guess of the sequence and/or the subunit location. In doing so, MISO-generated structures would inform users on their plausibility, as some combination of sequence and location may generate unphysical structures (e.g., crumpled pyranose rings, etc.). This capability highlights the role of MISO in validating and invalidating sequence and location hypotheses in the interpretation of SPM images of individual biomolecules.

### CREATE: Generating Monosaccharide Structures

In CREATE, sequence and location inputs are used to build the 3D structures of every monosaccharide at their respective (*x*,*y*) locations using the RDKit package.^46^ To account for diverse 3D structures that may be adopted by monosaccharides, especially those that possess flexible functional groups, we build MISO to generate a comprehensive library of 3D pyranose structures for any given monosaccharide. For every monosaccharide, MISO screens these generated structures by geometry, energy, and RMSD (root-mean-square difference) before outputting the most energetically favorable structure. MISO uses Cremer-Pople formalism to characterize the geometry of the pyranose structures^47^ and computes the energy of the pyranose structures by the classical force field implemented in RDKit (see Methods for details).

As an example, we show a case of *N*-acetylneuraminic acid (Neu5Ac) (Figure 3), which possesses a flexible alkyl chain (C7–C9) as well as rotationally flexible carboxy, hydroxy, and *N*-acetyl groups. For the Neu5Ac subunit, MISO generates a library of chairlike and boatlike structures along with varied geometries of the C7–C9 side chain. This example demonstrates the capability of MISO in allowing users to build the final 3D molecular structures by using a diverse selection of monosaccharide 3D structures.

**Figure 3:**
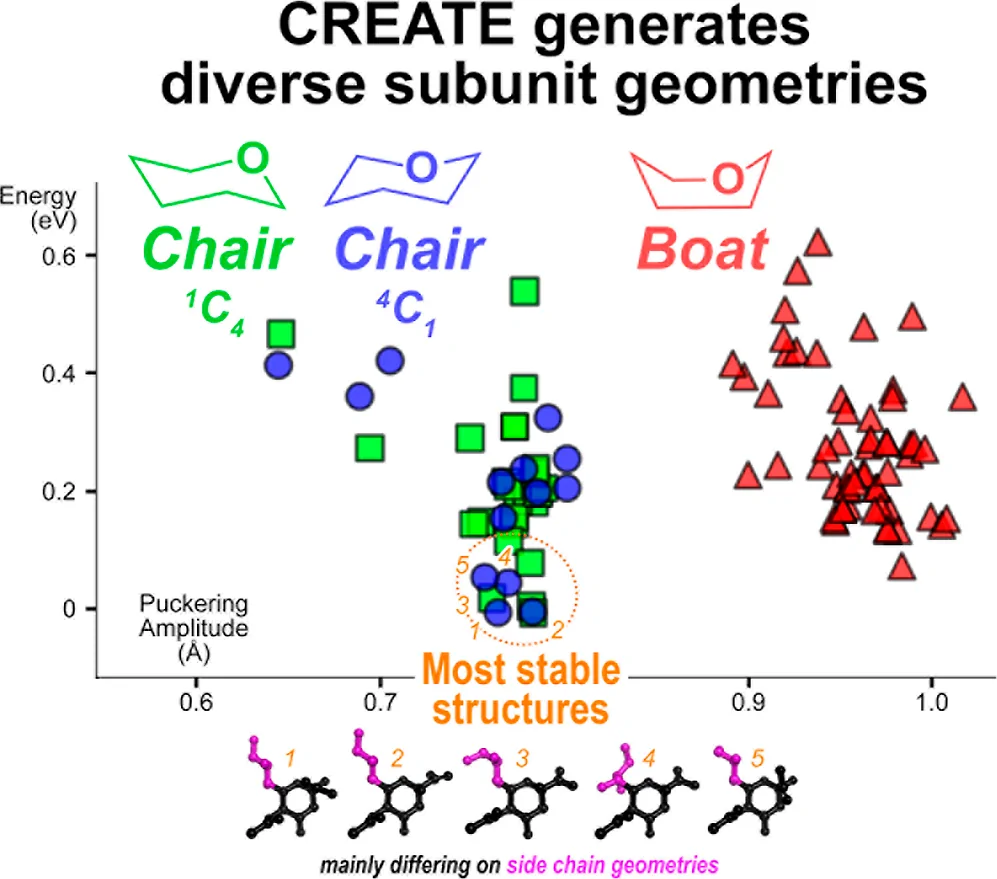
Diverse structures of *N*-acetylneuraminic acid (Neu5Ac) generated by MISO-CREATE. A plot of potential energy against the Cremer-Pople puckering amplitude (Q) shows the distribution of 100 MISO-generated structures of Neu5Ac. Marker shapes denote conformational families: circles (^4^C_1_ chair), squares (^1^C_4_ chair), and triangles (boat) (see Method for definitions). Five Neu5Ac geometries with the lowest energies are visualized, showing the Neu5Ac mainly with varied side chain geometries. The MMFF94 force field used here is benchmarked to be accurate to ∼1 kcal/mol (∼43 meV) against high-level ab initio calculations.^48^

### ROTATE: Optimizing Monosaccharide Orientations

In ROTATE, MISO optimizes the 3D orientation of every monosaccharide in its respective location according to the connectivity defined in the input. We implement this optimization by performing rigid rotation on every monosaccharide around its respective center-of-mass (or geometric center) pivot with the goal of bringing the atoms linking the monosaccharides to be within a chemically relevant distance (see Methods for details in choosing pivots). As such an alignment problem is mathematically equivalent to Wahba’s problem,^49^ we employ the QUEST (Quaternion Estimator) algorithm^44^ to provide a fast analytical method to find the rotation that best aligns a monosaccharide to its bonding partners (see Methods for implementation and parametrization details).

As an example, we show in Figure 4 step-by-step how MISO optimizes the 3D orientation of every monosaccharide in a glycan by using QUEST. Starting from a root mannose (Man) monosaccharide, MISO performs QUEST to find the best rotation matrices for the root mannose and its three partners so that the relevant O atoms are brought within chemically relevant distance. Specifically, QUEST would bring the O1 of the root mannose close to the O4 of the neighboring *N*-acetylglucosamine (GlcNAc) as well as the O3 and O6 of the root mannose to the O1 of the respective mannose neighbors. The completion of this first QUEST step initiates a new QUEST step between different monosaccharide groups, which would eventually yield the globally optimized orientation for every monosaccharide in the glycan.

**Figure 4:**
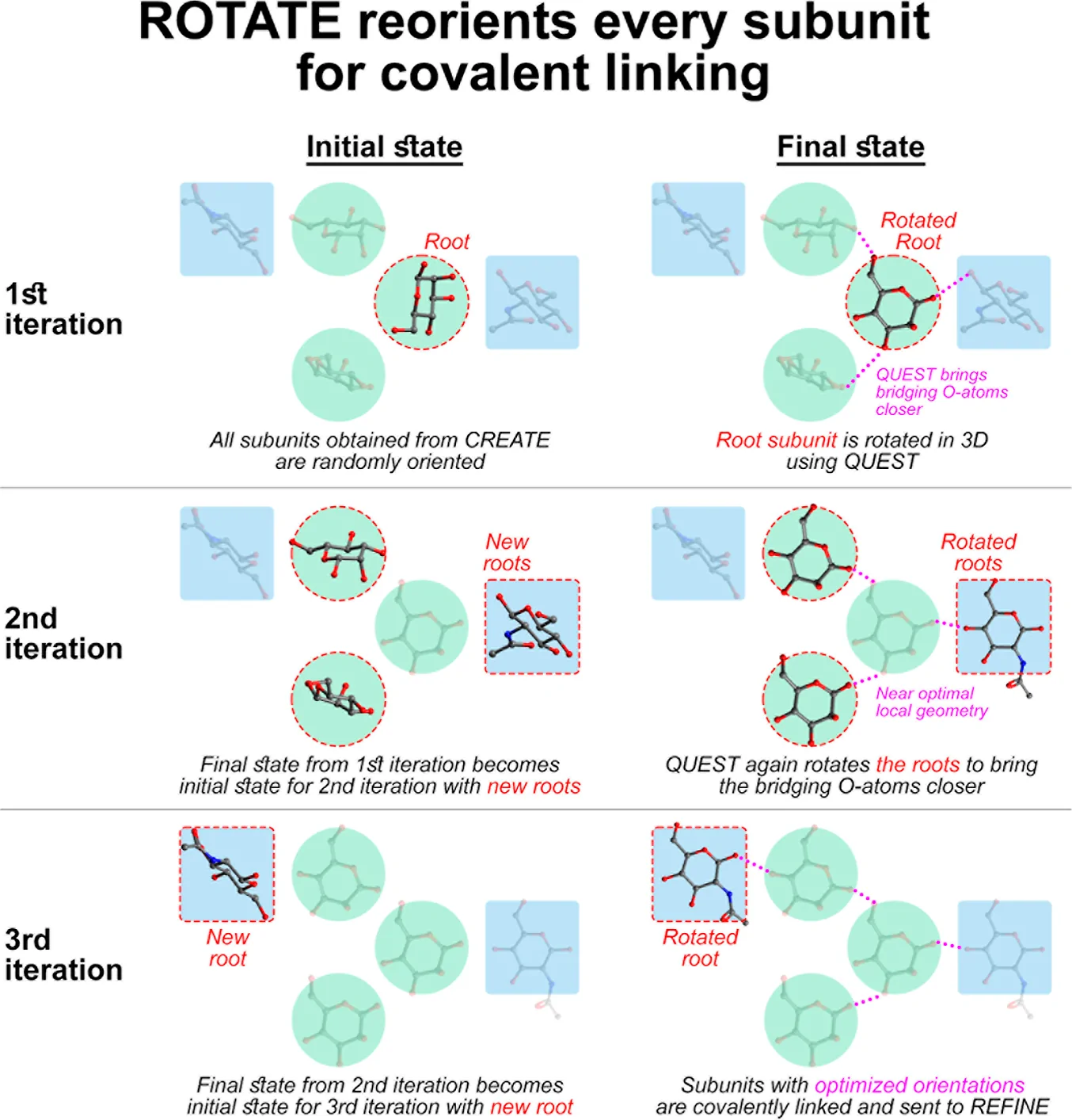
Optimizing individual monosaccharide orientations using QUEST in MISO-ROTATE. Step-by-step illustration on how QUEST iteratively estimates rotations for every monosaccharide subunit, starting from a user-defined root molecule and propagating outward.

To manage the possibility of multiple solutions from QUEST, we have implemented constraints and a scoring framework to assess the resulting solutions (see Methods for details). First, MISO evaluates every QUEST solution against two geometric tests: the ring clearance test (preventing subunit-bridging bonds between relevant O atoms from passing any pyranose ring) and the bond intersection test (preventing subunit-bridging bonds from crossing one another). Afterward, MISO assesses the QUEST solutions for steric clashes using van der Waals (vdW) radii, where a solution is accepted only when no (or minor) steric clash is detected. In a case where all QUEST solutions fail the tests above, MISO iteratively rotates every monosaccharide in a brute-force manner to search for an optimized global monosaccharide orientation. Overall, the strategy implemented in MISO leverages the speed of analytical methods, such as QUEST, while at the same time ensuring robustness through exhaustive brute-force sampling when analytical methods fail.

The QUEST solutions that pass the geometric tests above are subsequently scored (lower means better) by

1 $$score=\mathrm{d̅}\times w_{d}+P_{collision}\times w_{c}$$

where *d̅* is the average distance of all subunit-bridging bonds weighted by *w*_d_ and *P*_collision_ is a measure of vdW steric clashes weighted by *w*_c_. In eq 1, we have set *w*_c_ to be larger than *w*_d_ because severe steric overlaps are harder to remove in subsequent optimization steps than suboptimal bond lengths. From all the scored QUEST solutions, MISO favors solutions with low scores.

Finally, MISO employs the ROTATE pipeline to generate 3D structural candidates for the downstream analysis. First, MISO considers two monosaccharide configurations, differing by which side of the pyranose ring faces “upward” (+*Z* direction). This consideration effectively doubles the rotational search space of the QUEST per monosaccharide subunit. Next, once optimized solutions are found for every monosaccharide subunit, MISO constructs variants using a systematic variation strategy: the first candidate uses the best (rank-1) rotation for all subunits, while subsequent candidates vary one subunit at a time to its next-best rotation (rank-2, rank-3, etc.), holding all other subunits at rank-1. This generates up to *N* structurally distinct candidates that differ in local monomer orientations while maintaining overall assembly feasibility, where the value of *N* can be adjusted by users.

Overall, in ROTATE, the combination of QUEST analytical efficiency, geometric validation, exhaustive sampling fallback, and systematic structure generation provides a robust framework for sequential polymer-like assembly that balances speed with reliability and structural diversity—an approach that may prove valuable for other computational structure determination problems involving constrained monomer positioning.

### REFINE: Optimizing Structures by Classical MD

In REFINE, MISO processes the structural candidates from ROTATE, which may still contain imperfections (e.g., strained bonds), into fully relaxed structures that are ready to be compared with the corresponding SPM image. First, for every candidate structure from ROTATE, MISO builds the glycosidic bonds between the monosaccharide subunits, as well as covalent modifications to the glycan structures, such as lipid chains or polypeptide chains (see Supporting Information for details). Next, these additions are followed by a series of force field MD-like optimizations via RDKit^46^ to yield fully relaxed structural candidates for final output (Figure 5) through a three-step protocol (see Methods for details):

1. Initial relaxation was used to relieve local strain, with the pyranose ring geometries frozen.
2. The molecular structure was flattened onto the *xy* plane.
3. Final unconstrained relaxation was performed.

**Figure 5:**
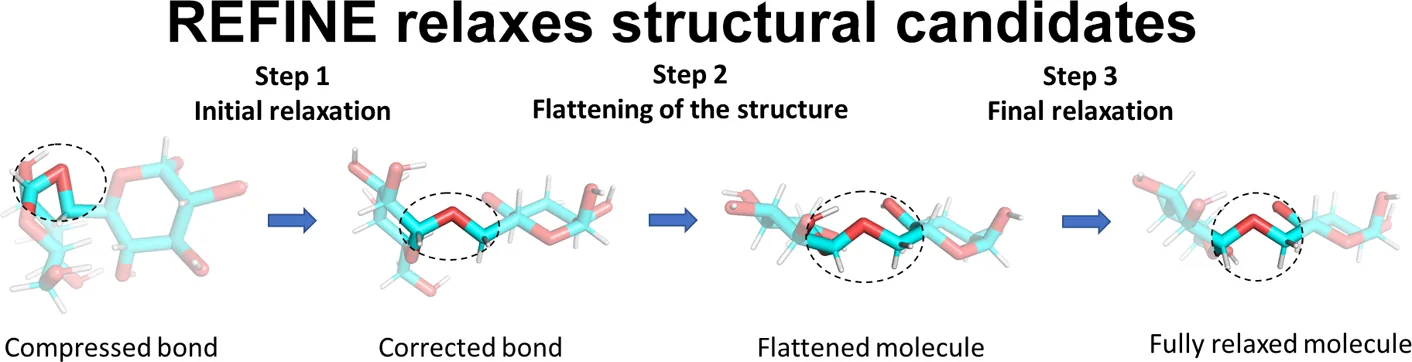
Structural relaxation in MISO-REFINE. Step 1 relaxes the structure with the pyranose rings frozen. Step 2 flattens the molecule using MD modified with biasing forces that compress the molecule from below and above (see Methods for details). Step 3 relaxes the molecule without any constraint for final output.

We implement step 2 in MISO to access geometries that maximize the contact between the molecule and the underlying surface (see Methods for implementation details). We justify this based on the notion that at present direct SPM imaging is used to visualize unfolded molecules on the surface, where individual subunits are deliberately made to maximize their contact with the surface to achieve the best imaging stability. We accomplish such molecular flattening by performing MD added with biasing forces, namely, a gravity-like downward force as well as an elevator-like upward force—which both work in tandem to flatten the molecule on the *xy* plane. Notably, the flattening of molecular structures is found to lower the diversity of the structural candidates, as shown by a significant change in the RMSD profile before and after the flattening procedure. As shown in Figure 6 for GM3 glycolipid, the RMSD profile of the structural candidates preflattening appears to be diverse, while the RMSD profile of the postflattening structures appears to be more uniform, indicating that few structural candidates converge into a single structure candidate. This result highlights the ability of flattening the structural candidates to narrow the MISO solution space into fewer classes.

**Figure 6:**
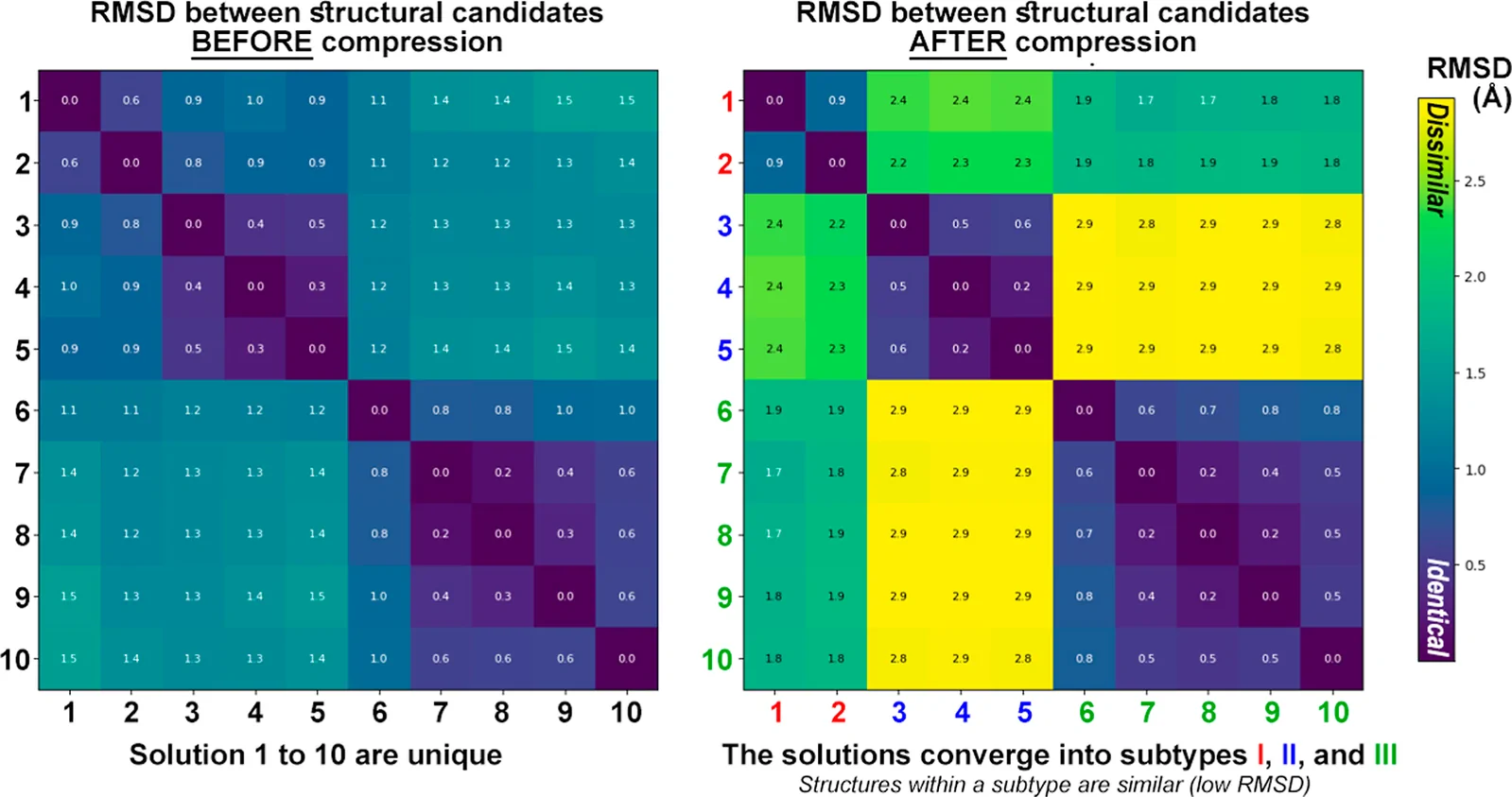
RMSD evolution before and after MISO-REFINE. Pairwise RMSD for ten conformations of the GM3 glycolipid case after initial optimization (Step 1, left) and after compression (Steps 2–3, right). Comparison of the RMSD shows convergent structure optimization: the ensemble partitions into 3 distinct conformational subtypes, indicating that the program identifies stable energy minima. The discrete clustering pattern, rather than a continuous conformational distribution, reflects restricted torsional degrees of freedom imposed by glycosidic linkage energetics and geometric constraints of surface adsorption.

Taken altogether, the three-step protocol in REFINE is designed specifically for high-throughput SPM-image-to-structure model building, where (i) 3D structures are generated to account for the observed molecular topography rather than energy minimization and (ii) computational efficiency is prioritized over rigorous sampling.

### MISO Application

By deploying MISO to real data, we show MISO-generated structures to be in excellent agreement with the manually solved structures of glycans and glycoconjugates in our previous work^9^ (see Methods for brief experimental details). We show MISO satisfactorily reproduces the DFT-verified molecular structures of a simple glycopeptide (Glcβ1–4Glc linked to Asn-Phe-Ala tripeptide, Figure 7A), GM3 ganglioside glycosphingolipids (Figure 7B), an 8mer heparan sulfate glycosaminoglycans (Figure 7C), and an egg yolk sialoglycopeptide derivative (Figure 7D). We note that differences between MISO structures and DFT-verified structures are to be expected, as quantum-mechanical molecule–surface interactions are not included in MISO, unlike in DFT. Hence, we highlight qualitative agreement in subunit geometries between MISO structures and DFT-verified structures, thereby underscoring MISO as a tool that eliminates manual model building of biomolecules on the surface.

**Figure 7:**
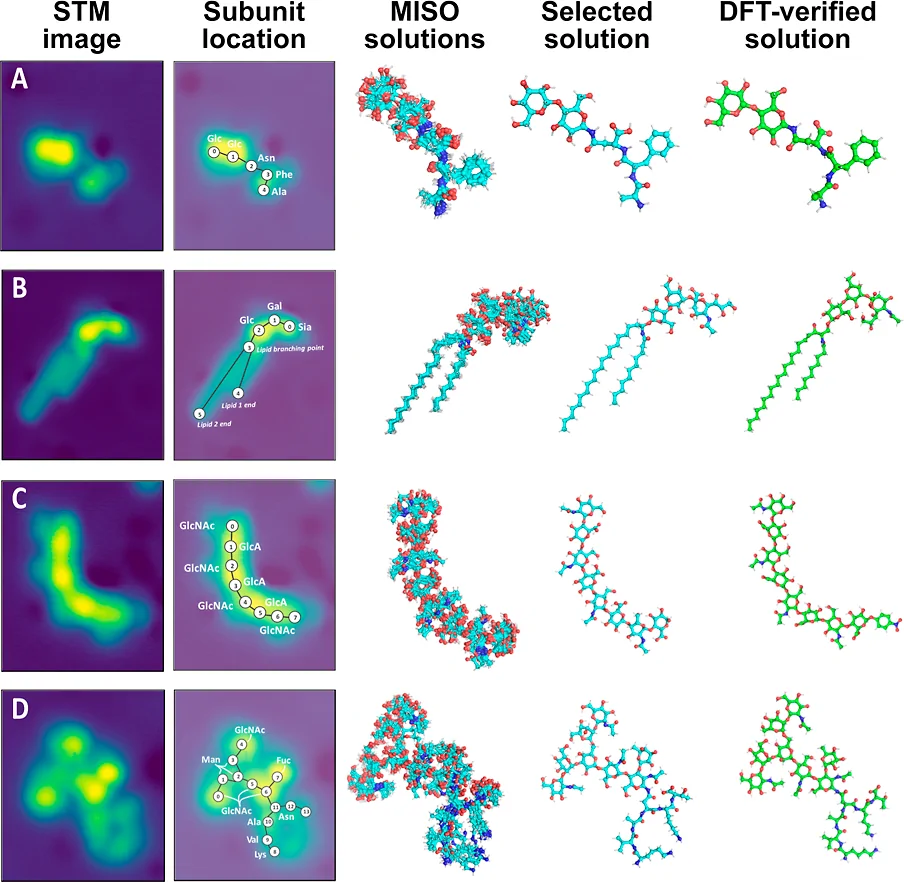
Applying MISO into real SPM image of glycans and glycoconjugates. MISO deployed onto SPM images of a synthetic glycopeptide (A), a GM3 glycolipid (B), a glycosaminoglycan (C), and a sialoglycopeptide (D) gives satisfactory agreement between the MISO-generated structures and the DFT-verified manually solved structures. The molecules were deposited intact and imaged by STM on the Cu(100) surface (see Methods for details).

We further show the use of MISO as a model-building tool in eliminating user hypotheses that lead to unphysical structures. Using the sialoglycopeptide as the example (Figure 7D), we use MISO to build 3D models initiated with alternative subunit connectivity (Figure 8, hypothesis 1) or subunit location (Figure 8, hypothesis 2). For hypothesis 1, the MISO-generated structures for the alternative hypotheses show the subunit to move significantly from the hypothesized subunit location to prevent the generation of highly strained structures (e.g., crumpled pyranose rings), which leads to rejection of the hypothesis. For hypothesis 2, alternative subunit position leads to MISO-generated structures with extremely bent C–O–C glycosidic bonds (∼130°), which leads to rejection of the hypothesis. These results demonstrate the use of MISO as a first-line hypothesis tester without vast time investment via the automated building of the 3D molecular model.

**Figure 8:**
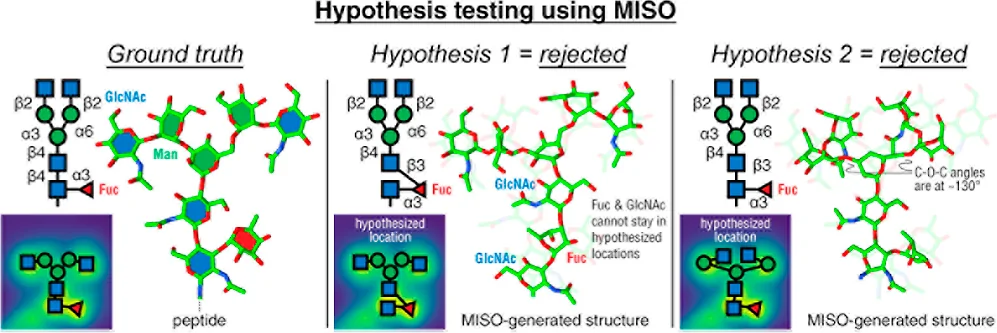
Using MISO for hypothesis testing. MISO is used to build 3D molecular models with different user-hypothesized primary structures of a sialoglycopeptide, which allows users to invalidate some primary structures due to unphysical structures (e.g., extremely stretched and bent glycosidic bond). Hypothesis 1 features alternative subunit connections, while hypothesis 2 features alternative subunit locations. These hypotheses lead to MISO-generated structures that lead to rejections of these hypotheses. For hypothesis 1, the structure shows Fuc and GlcNAc subunits moved significantly from the hypothesized locations; while for hypothesis 2, the structure shows the presence of extremely bent glycosidic bonds. The ground truth structure is obtained from the DFT relaxation followed by a good agreement of its STM simulation with the experimental image.

MISO success in automated model building for small to large biomolecules (from 89 to 286 atoms) underscores its scale-up potential to tackle much larger molecules, such as glycoproteins, RNAs, or polysaccharides. In assessing the scale-up potential of MISO, our analysis shows that 99% of the total runtime is dominated by the force field optimization stage, whereas preceding steps (e.g., structure generation, chain assembly, QUEST optimization, and structure export) contribute only tenths of a second or less. Specifically, the molecular flattening step (step 2) in REFINE is found to be the main bottleneck, consuming 98.4% of the total optimization time, while constrained minimization (step 1) and final minimization (step 3) only contribute 1.4% and 0.2%, respectively. To address this bottleneck, we have enabled manual adjustment of the iteration taken in step 2 of REFINE to allow user control of the runtime: fewer steps reduce computation time at the cost of less thorough geometry exploration, while more steps improve structural quality at greater computational expense.

Further bottleneck analysis in step 2 of REFINE shows for all cases (Table 1) that the MMFF94 force field evaluation dominates the runtime. This bottleneck imposes a time cost of 29 ms per MD step for the smallest molecule (89 atoms) to 597 ms per step for the largest (286 atoms), thus showing a 21.5-fold increase in runtime for a 3.2-fold increase in the atom count. Fitting a power law to force field evaluation time against the atom count yields a scaling exponent of approximately 2.6 on atom count, consistent with the quadratic complexity expected from MMFF94 pairwise noncovalent interactions under a full atom-pair evaluation scheme.^50,51^ These results establish that the runtime is governed almost entirely by the superquadratic scaling of MMFF94 force evaluation with respect to the total atom count. Future optimization efforts could therefore focus on the force evaluation step, such as enforcing cutoff-based noncovalent interaction schemes, neighbor list implementations, or replacing MMFF94 with a faster approximate force field while retaining full MMFF94 accuracy.

**Table 1:** | Runtime Breakdown for Step 2 of REFINE (1500 Steps Each)

Molecule	Total atom (excl. H)	Total atom (incl. H)	Pyranose count	Lipid chain count	Peptide length count	Total runtime (s)	Runtime per step (ms/step)
A	68	89	2	0	3	44.4	29.6
B	69	153	3	2	0	165.5	110.3
C	105	187	8	0	0	285.7	190.5
D	197	286	8	0	6	895.7	597.1

Since CREATE and ROTATE are computationally inexpensive when compared to REFINE, MISO also allows the possibility for users to inspect the optimized but unrefined structural candidates. This decoupled workflow allows efficient screening of the candidate pool, where the user first evaluates the structures in the candidate pool, before selecting which structures merit full or partial REFINE treatment, depending on the number of iteration steps in REFINE. This alternative design renders the MISO pipeline to be practical for generating a large structural ensemble, as the expensive REFINE step is applied only to selected, promising candidates rather than exhaustively to all generated structures.

To show the perspective of MISO, we show the use of MISO in building the 3D molecular structure of an unfolded RNaseB glycoprotein from our previous work^9^ (Figure 9). MISO is demonstrated to build molecular structures containing 2042 atoms from user-hypothesized subunit identity, connection, and location, illustrated as the protein backbone contour approximating the Cα position of every amino acid (yellow line, Figure 9) and as glycan structure approximating the position of every monosaccharide in the glycan (white line, Figure 9). Our benchmark of such automated model building shows that the structure-flattening step (Step 2 of MISO-REFINE) again the most expensive step in the MISO algorithm. The step dominates as much as 99.6% of the total time (31.8 h), costing ∼2 min per step for a total of 1000 steps, underscoring the need to optimize such a step (e.g., by GPU acceleration^52^) or by adopting machine-learning force fields (see below). The runtime, in the absence of a structure-flattening step while still applying force-field relaxation (Step 1 and 3 of MISO-REFINE), is cut down to ∼7 min to give 3D structures devoid of hard-sphere overlaps and all chemical bonds respecting usual VSEPR geometries (e.g., tetrahedral geometry for a Csp^3^-atom, and trigonal planar geometry for a Csp^2^-atom). The short runtime of ∼7 min to obtain a 3D model highlights MISO as a lightweight tool to quickly assess user hypotheses, which are particularly important when the users are operating live SPM imaging.

**Figure 9:**
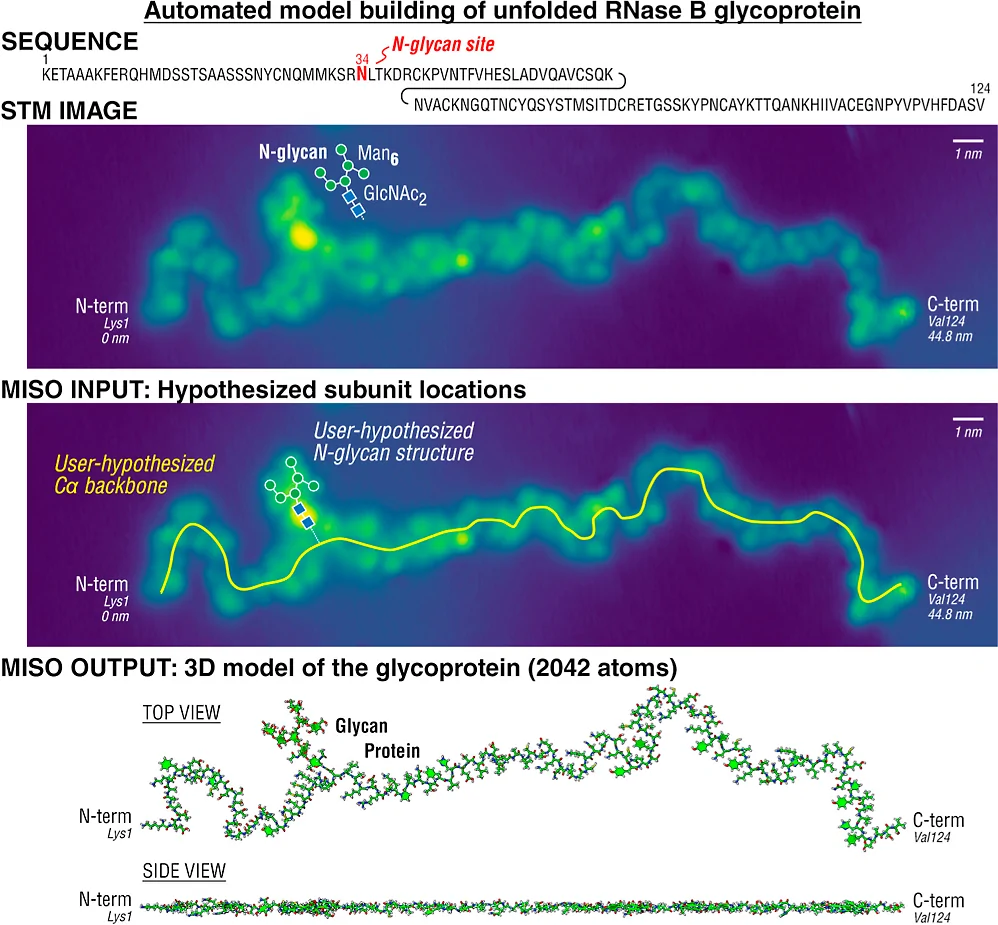
Using MISO to build a 3D model of unfolded RNaseB glycoprotein. MISO is used to automatically build a 3D molecular model of the unfolded glycoprotein. The input used is a set of (*x*,*y*) coordinates describing the protein Cα backbone (shown as a yellow line) and the glycan structure (shown as a white line). The Cα position of every amino acid is assumed to be equidistant from one another along the user-hypothesized backbone. The output structure shown is the lowest energy, flattened structure, ready for DFT relaxation and SPM image simulation.

### MISO Roadmap

While we have shown MISO successes in automating model building of glycans and glycoconjugates, we highlight several opportunities between MISO and a few contemporary machine learning (ML) approaches that would allow the SPM image-to-structure task to be realized in the long run. In addition, specifically targeting MISO toward glycoscience, we anticipate a fruitful synergy in incorporating a publicly accessible glycan structure, shape, and topology database into the MISO workflow.^53,54^

#### Accelerating Structural Explorations Using Machine-Learning Force Fields (MLFF)

Our present analysis shows force field evaluation in REFINE to be the main bottleneck of MISO, due to the evaluation of noncovalent interactions for every possible atom–atom pair. Still, a classical force field, at present, offers the best trade-off between speed and accuracy, especially for standard covalent geometries (i.e., a tetrahedron for C-sp3, trigonal planar for C-sp2, and so on). Surpassing this accuracy without costly ab initio methods poses challenges that limit how exhaustively MISO can explore the 3D structures of a system.

Here, we anticipate MISO would benefit from recent developments in MLFF.^55^ Particularly, recent works have shown MLFF trained on DFT data to reproduce near-chemical accuracy (1 kcal/mol or 43 meV) while retaining the computational efficiency required for large-scale 3D structural searches. For biomolecular systems, general-purpose MLFFs, such as SO3LR,^56^ demonstrate high accuracy for flexible organic molecules and peptides, underscoring their strong potential to address the geometrical diversity of glycans and glycoconjugates. On the other hand, to account for the molecule–surface interaction, models, such as MAD-SURF,^37^ are trained explicitly on molecule-metal adsorption systems and achieve chemical accuracy for molecule–surface interactions. We anticipate that incorporation of MLFFs into the MISO algorithm would enable automated model building for macromolecular systems (e.g., glycoproteins) a few hundred kDa in size, typical in molecular biology studies.

#### Scoring Structures Using ML-Accelerated Electronic Structure Methods

Another challenge in MISO concerns how MISO-generated structures should be evaluated against the SPM images obtained from experiments. At present, MISO-generated structures are subjected to DFT relaxation on the surface followed by SPM image simulation. However, the high computational cost of DFT calculations limits the number of MISO-generated structures that could be simulated and compared against the experimental SPM images.

Integrating ML-accelerated electronic structure methods to MISO offers a pathway to overcome this limitation without the need for any DFT calculations. Methods predicting electron density trained on DFT data^57,58^ use atomic coordinates to generate electron density, out of which SPM images could be simulated. In the context of simulating STM images, the use of ML electronic structure methods should eliminate the need to perform computationally expensive SCF loops in ab initio calculations. However, the resulting ML-generated electron density still needs to be diagonalized into eigenstates to approximate the local density of state needed for simulating STM images by the Tersoff-Hamann approach.^43^ Still, we anticipate that the use of ML-generated electron density would significantly cut down the computational cost needed to generate simulated SPM images and enable high-throughput comparison of MISO-generated structures with the experimental SPM images.

#### Automating Image-to-Structure Analysis Using Computer Vision

In envisioning MISO to help realize image-to-structure interpretation in SPM images, we specifically envision MISO to be paired with tools capable of recognizing molecules on surfaces directly from a live SPM data feed and without any a priori knowledge of their structures, to suggest possible structures for the observed molecules. Realizing this would be one of the key steps to elevate SPM imaging into a high-throughput single-molecule analysis platform for biomolecules.

We expect that automating such image-to-structure tasks requires pairing MISO with tools that perform in real-time image segmentation for molecules on the surface—that is, the ability to recognize specific objects from a live image feed, similar to object detection by modern cars. Several models employing computer vision techniques^34,35^ have shown promise in detecting small molecules on surfaces, with few models showing promising progress in interpreting molecular features into molecular structures.^38,39^

Along this direction, we anticipate two developments for the MISO. The first involves the use of MISO to perform model building on batches of biomolecular SPM images, where we anticipate MLFF and ML-accelerated electronic structure methods to play key roles here. Then, we trained a convolutional neural network (CNN) to interpolate the SPM images into their corresponding MISO-solved molecular structures. We anticipate that this CNN would be instrumental to perform image-to-structure operations to quickly suggest molecular structures from a molecular appearance, as well as structure-to-image operations to quickly simulate SPM images from a molecular structure for scoring purposes without the use of DFT. Preliminary work along this direction has shown promise for further development.^59^

### Summary and Outlook

Summary and Outlook: to sum up, we demonstrate MISO as a systematic workflow to generate 3D structures of glycans and glycoconjugates from user-defined hypotheses about subunit identity, connection, and location in real space. MISO is designed to be modular with the CREATE, ROTATE, and REFINE modules to create 3D structures of monosaccharide subunits in the target glycan or glycoconjugates and orient these structures before stitching and relaxing them together using classical MD simulations. We developed MISO with the main goal of reducing the need for manual molecular model building needed in the SPM image interpretation task, which becomes practically infeasible for large polymer-like molecules. While we recognize that manual interpretation is still able to produce valid structural solutions, manual interpretation relying on chemical intuition, although valuable, may not systematically explore all possible structural candidates and may overlook alternative geometries that provide equally good or superior fits to experimental data. Automated approaches, such as MISO, enable exhaustive structural searches to potentially reveal structures not initially considered—and, at the same time, reduce the overall time required for model building in the SPM image interpretation work.

We chart the MISO roadmap in addressing the challenge of automated model building for SPM imaging of biomolecules by highlighting potential integration of ML into the MISO pipeline, starting from MLFF-driven structural relaxations, ML-accelerated electronic methods for structure scoring, and a CNN-powered engine for fast image-to-structure and structure-to-image processes. These developments, when coupled with HPC infrastructure and an interactive graphical user interface, position MISO as a key engine that powers SPM imaging as an analytical platform for single-molecule analysis for complex (bio)­molecular systems.

### Methods

MISO was executed on a high-performance computing (HPC) cluster node equipped with an Intel Xeon CPU E5 v3 @ 2.6 GHz (2 threads per core) using 4 parallel tasks.

Glycans and glycoconjugates shown in Figure 6 were previously reported in ref 9. Briefly, these molecules are electrosprayed, mass-selected, and deposited on the surface using the home-built electrospray ion beam deposition technique (ESIBD). The molecules were deposited at a clean Cu(100) surface held at 120 K in UHV with energies as low as 18 meV/atom, which is well below the energies associated with soft landing (∼40 meV/atom). Prior to the deposition, the Cu-surface was cleaned by repeated cycles of Ar-ion sputtering and annealing. The deposited molecules were imaged by a Scienta Omicron LT STM (Fermi SPM) at 11 K with a constant current mode at a bias voltage of +0.3 V and tunneling current set point of 1 pA. As mentioned in ref 9, glycans and glycoconjugate structures were solved by manually exploring and building molecular models in various geometries, which were subsequently subjected to DFT relaxations on surface and STM image simulation. The STM image was considered interpreted as these DFT structures gave a simulated STM image that matches with the experimental STM image, as shown in ref 9.

#### Create

MISO uses CREATE to generate the 3D structure of the monosaccharides in a glycan or glycoconjugate, presently limited to hexoses in a pyranose conformation.

For each pyranose monomer, we first number the C-atom in the pyranose ring to ensure consistent labeling and facilitate accurate glycosidic linkage formation during later assembly. MISO numbers the C-atoms based on the O-atom in the pyranose ring, where, of the two C-atoms connected to the pyranose O-atom, the one with more O-atom neighbors is designated C1, with subsequent C-atoms numbered sequentially. From the C-atom numbering, directional vectors are computed from the monomer center (*r⃗*_center_) to each labeled carbon position. These vectors, d⃗ _C*i*_ = *r⃗*_C_*i*__ – *r⃗*_center_ (*i* = C-atom index), encode the monomer orientation and serve as references in QUEST. By default, the monomer center is set to the geometric center of the monomer (i.e., the average of all atoms in the monomer), but users may choose the center of mass of the monomer as well. The latter performs better for the monomer with significant mass asymmetry (e.g., Neu5Ac); while the former performs better for the monomer with high symmetry (e.g., Glc).

Ring conformations are systematically analyzed using Cremer-Pople puckering parameters.^47^ Since MISO generates pyranose conformers with arbitrary spatial orientations via RDKit, MISO establishes a reference frame to define the pyranose ring plane. For each pyranose conformer, MISO centers the pyranose ring at the average coordinate of the six atoms in the pyranose ring and applies singular value decomposition (SVD) to the six ring atoms to determine the vector **n** normal to the ring plane (i.e., from the eigenvector corresponding to the smallest eigenvalue). Out-of-plane displacements *z*_*j*_ for each atom *j* are calculated as projections onto these normal and puckering coordinates *q*_*m*_ (*m* = 2, 3, 4, 5) are computed via the standard Cremer-Pole Fourier transformation. From these, three rotationally invariant parameters are derived: total puckering amplitude $Q=\sqrt{\sum_{m=2}^{5}q_{m}^{2}}$ (in Å), polar angle θ = arccos­(*q*_3_/*Q*) (in a range of 0–180°), and pseudorotation phase ϕ = arctan2­(*q*_4_,*q*_2_) (in a range of 0–360°). Conformational classification proceeds hierarchically based on established geometric criteria.^47^ Chair conformations are identified when θ < 15° (^4^C_1_-like) or θ > 165° (^1^C_4_-like), while boat/skew-boat geometries occur at 75° ≤ θ ≤ 105°, half-chair conformations at 35° ≤ θ ≤ 65° or 115° ≤ θ ≤ 145°, and envelope conformations at 15° ≤ θ ≤ 35° or 145° ≤ θ ≤ 165°. Envelope subtypes are further distinguished by ϕ-angle ranges in 60° increments, while planar geometries are identified when *Q* < 0.1 Å. This automated classification enables rapid filtering of conformational ensembles based on ring pucker geometry.

To eliminate redundancy within the generated conformational ensemble, structures are compared by the RMSD of all atomic coordinates, excluding H atoms. Conformers exhibiting RMSD values below a defined threshold are considered equivalent and are clustered together. From each cluster, only the structure with the lowest computed energy was retained for subsequent analysis. This dual filtering strategy, combining Cremer-Pople-based geometric classification with RMSD-based redundancy elimination and energy-based selection ensures that the final conformational ensemble represents energetically favorable and geometrically distinct ring-puckering states.

Following conformer generation and filtering, each selected conformer is positioned at its (*x*,*y*) location, as defined by the MISO input. Each monomer is translated as a rigid body to preserve all its internal coordinates (i.e., bond lengths, angles, and dihedral angles) established during conformer generation and ensure that the conformational properties characterized via Cremer-Pople analysis are maintained during spatial assembly.

#### Rotate

Following monomer assembly at their respective locations, MISO uses ROTATE to orient every monomer structure for their subsequent covalent linkage. Given that each monomer is treated as a rigid body parametrized with directional vectors d⃗ _C_*i*__ defined in CREATE, QUEST finds the rotation matrix *A* that minimizes the weighted alignment error l(A)

$$l\left(A\right)=\frac{1}{2}\sum_{i=1}^{n}\alpha_{i}|\left|w_{i}-Av_{i}\right|{|}^{2}$$

where *v*_*i*_ are unit vectors of *d*_C_*i*__, *w*_*i*_ are unit vectors from the monomer center (*r⃗*_center_) toward target bonding positions on neighboring monomers (see Figure S1 for vector definition), and α_*i*_ are normalized real-number weights (∑_*i*_ α_*i*_ = 1) that prioritize bonds by importance. To explore different prioritization strategies, MISO generates multiple QUEST solutions using systematically varied weight distributions, such as

- Primary bond emphasized (e.g., α_1_ = 3, others = 1)
- Equal weights (e.g., α_*i*_ = 1 for all bonds)
- Primary bond strongly emphasized (e.g., α_1_ = 5, others = 1)
- Each additional bond emphasized in turn (e.g., α_*i*_ = 3, others = 1)

After the relative weight assignment, all weights are normalized to unity. This approach explores the trade-off space between satisfying different bonding constraints, as each weight distribution represents a different optimization objective that may yield geometrically valid solutions even when the default weighting fails.

QUEST reformulates this problem as a single eigenvalue calculation. The algorithm constructs a 3 × 3 rotational orientation (attitude) profile matrix *B*

$$B=\sum_{i=1}^{n}\alpha_{i}w_{i}v_{i}^{T}$$

From *B*, auxiliary quantities capturing different geometric aspects of the alignment are computed

$$\begin{array}{l} \sigma =tr\left(B\right)=B_{11}+B_{22}+B_{33}\\ S=B+B^{T}\\ Z=\left[\begin{array}{l} B_{23}-B_{32}\\ B_{31}-B_{13}\\ B_{12}-B_{21} \end{array}\right] \end{array}$$

These are combined into a 4 × 4 matrix *K* that reformulates the 3D rotation problem in quaternion space

$$K=\left[\begin{array}{ll} \sigma & Z^{T}\\ Z & S-\sigma I_{3} \end{array}\right]$$

The optimal rotation is encoded in the eigenvector q* = [*q*_0_, *q*_1_, *q*_2_, *q*_3_]^*T*^ corresponding to the largest eigenvalue

$$Kq^{*}=\lambda_{\max}q^{*}$$

This quaternion is converted to a 3 × 3 rotation matrix *A* that preserves distances and angles (a proper orthogonal transformation), which is then applied to rotate the monomer’s atomic coordinates into optimal alignment with the target structure.

QUEST is performed on every monomer to orient the individual monomers in a globally optimized state. The resulting orientation is subjected to geometric tests, as detailed below:

##### Ring Clearance

Bonds formed between monosaccharides are prohibited from crossing through the rings of any neighboring monosaccharides. For each bond, the algorithm checks whether the bond trajectory intersects any potentially obstructing ring planes within a safety margin (1.2× ring radius) that accounts for atomic radii and structural flexibility. The ring plane is calculated using SVD to determine the best–fit plane through all ring atoms, which properly handles nonplanar ring conformations such as chair, boat, or envelope puckering common in pyranose rings. Rotations that violate ring clearance are immediately rejected.

##### Bond Intersections

To prevent topologically invalid structures, the algorithm checks whether bonds formed between monosaccharides would intersect with any existing bonds when projected onto the *xy* plane. For single-bond formation, the new bond is tested against all existing glycosidic linkages. When a monomer forms multiple bonds simultaneously, all pairs of new bonds are additionally checked for mutual intersections. Any rotation producing bond intersections is rejected, as such configurations cannot be resolved by subsequent force field optimization.

##### Steric Clashes

Overlaps between nonbonded atoms are detected using standard vdW radii with a permissive clash factor. A tolerance allows minor overlaps that are resolvable by subsequent force-field optimization. Unlike ring clearance and bond intersections (which trigger immediate rejection), steric clashes contribute to solution scoring rather than disqualification, as minor overlaps can be resolved during geometry optimization.

When QUEST fails to solve the global optimization problem, MISO performs an iterative sampling of rotation for every monosaccharide, where up to *N* quasi-uniformly distributed rotations are generated by selecting rotation axes via Fibonacci sphere point distribution and pairing each axis with a random rotation angle. This produces additional candidate rotations exploring the complete rotation space without an alignment bias. Uniform sampling continues until at least five valid rotations are found or the sampling budget is exhausted, whichever comes first.

#### Refine

Following global orientation optimization in ROTATE and covalent stitching of the monomers, the resulting structures are passed to REFINE for structural relaxation using a classical force field. Here, we detail the three steps of structural relaxations implemented in REFINE:

##### Step 1: Initial Relaxation

The structure is subjected to energy minimization using the MMFF94 force field, implemented via RDKit. This establishes a chemically plausible starting point with correct bond lengths, angles, and ring geometries for all subunits. Ring geometries are preserved through the force field, where bond stretching potentials maintain ring edge lengths, angle bending terms preserve ring angles, and improper torsions favor chair conformations over distorted geometries. To maintain the monomer locations in their initial locations during the structural relaxation, we add distance constraints using the RDKit MMFFAddDistanceConstraint function to the monomers separated by more than 10 Å, thereby maintaining the monomer–monomer distances within ±10% of their initial values. These constraints cause the structural relaxations to be mainly operative for the (ϕ,ψ) torsions between the monomers.

In MISO, the MMFF94 minimizer performs 4 conjugate gradient steps followed by an update to the coordinates—for a total of 1500 minimization steps. We use multiple short minimization cycles instead of a single long minimization cycle to improve convergence stability for constrained systems by allowing gradual relaxation rather than large conformational jumps. The relatively small number of steps per cycle prevents the minimizer from finding deep local minima that might violate the spatial constraints.

##### Step 2: Compression with Biasing Forces

Using the output structure from step 1 above, MISO explores the molecular conformation toward structural states observed experimentally where molecules tend to maximize their surface contact area. We accomplish this through a basin-hopping-inspired protocol combined with biasing forces that compress the molecule into a nearly flat state that strongly resembles its surface-adsorbed state. Here, the biasing forces consist of (i) gravity-like downward force and (ii) elevator-like upward force—which when both are operative would compress the molecules from below and above. In its MISO implementation, Step 2 consists of alternating steps between structural compression with biasing forces (i.e., the MD-based perturbation step) and structural relaxation (i.e., the relaxation step). We note that this method is a structure generation protocol, where we seek low-energy structures consistent with the experimental observation.

Biasing forces: for (i) gravity-like downward force, the gravity-like downward force acting on each atom *i* depends on its z-coordinate (*z*_*i*_) as follows

$$F_{i,z}^{grav}=\{\begin{array}{l} -m_{i}g,z_{i}>0\\ 0,z_{i}\leq 0 \end{array}$$

where *g* is a baseline downward force. For (ii) elevator-like upward force, we implement a repulsion between the atoms and an *xy*-plane that slowly moves up along the + *z* direction, as follows

$$F_{i,z}^{elev}=\{\begin{array}{l} k{\left(z_{slab}\left(t\right)-z_{i}\right)}^{n}ifz_{i}<z_{slab}+\Delta \\ 0ifz_{i}\geq z_{slab}+\Delta \end{array}$$

where *k* is a force constant, *n* = 2 creates quadratic repulsion in the transition zone (Δ = 1.5 Å above the slab) in which atoms with *z*-coordinate below *z*_slab_(*t*) + Δexperience repulsion away from the slab to keep all atoms above the slab. The slab rises monotonically in fixed increments (0.05 Å every *N* steps) until all atoms satisfy *z* < *z*_ceil_ = 2.2 Å.

In our protocol, the slab potential serves as a purely structural bias purpose to achieve the surface-adsorbed geometry in a fraction of the simulation time required by unbiased MD. This work is analogous to the computational Langmuir trough, in which a moving barrier compresses surface-active molecules at the air–water interface, driving transitions from disordered 3D configurations to ordered 2D surface-adsorbed geometries.^63^ Conceptually, the rising slab is also analogous to steered MD (SMD),^64^ in which a time-dependent external force drives a structural transition along a predefined coordinate.

In the MD implementation, the total force on each atom *i* is

$$F_{i}^{total}=F_{i}^{FF}+F_{i,z}^{grav}+F_{i,z}^{elev}$$

where **F**_*i*_^FF^ is the MMFF94 force field contribution, *F*_*i*,*z*_^grav^ is the gravity-like downward force weighted by the SPM image, and *F*_*i*,*z*_^elev^ is the rising slab. Repulsive floor and ceiling potentials confine atoms to the compression zone, preventing atoms from escaping the *xy*-plane over the target molecular thickness.

The molecular structure is compressed for 10 MD steps followed by 50 iterations of MMFF94 energy minimization, followed by velocity reset to small random values drawn from *N*(0, 0.01) Å fs^–1^. The periodic minimization every 10 MD steps relieves compression-induced strain before the next slab increment. Without this minimization, the slab would induce bond distortions faster than the force field can accommodate, thus leading to strained or broken geometries. In addition, the minimization step maps each MD perturbation onto the nearest local minimum of the force field, preserving the basin-hopping character of the search. The velocity reset prevents accumulation of kinetic energy and ensures that each mini-trajectory starts from a thermally accessible state. Unlike strict basin hopping, we omit the Metropolis acceptance criterion; this is justified by the nonequilibrium character of the slab compression potential, which changes the Hamiltonian at each step and renders a fixed-energy acceptance criterion thermodynamically inconsistent.^65^

Basin hopping: the alternating perturbation and minimization cycles follow the basin-hopping framework of Wales and Doye,^66^ in which the potential energy surface (PES) is transformed into a step function connecting local minima. This transformation enables efficient exploration of conformational space by accepting uphill moves between basins while rejecting downhill moves within the same basin, effectively eliminating trapping in high-energy local minima. We extend this framework by using short Langevin MD trajectories as the perturbation mechanism rather than random Cartesian displacements, an approach termed minima hopping and developed independently by Goedecker^67^ and applied to biomolecular systems by Schug et al.^68^ and Schönborn et al.^65^ The key advantage of MD-based perturbations over random displacements is that MD inherently respects bond geometry, steric constraints, and local force-field topology, generating physically realistic trial configurations rather than high-energy distorted structures. This is particularly critical for compressed glycans, where the combination of rigid pyranose rings, restricted glycosidic torsions, and external flattening forces creates a highly constrained conformational landscape. Random Cartesian perturbations would violate bond lengths, create severe steric clashes with the rising slab, or distort ring geometries—all leading to immediate rejection.

Short MD trajectories explore accessible conformational rearrangements within the compressed geometry, primarily through rotations around glycosidic linkages that accommodate the surface constraint while maintaining chemically valid bond lengths and angles, especially ring puckering states. This localized exploration maintains the basin-hopping character of the search: each MD perturbation samples nearby configurations consistent with the current compression level without diffusive drift, allowing the protocol to identify adsorption conformations through systematic testing of glycosidic bond orientations rather than high-energy structural distortions. Additionally, to ensure pyranose rings maintain chemically valid chair conformations throughout compression, we implement periodic geometry validation. For every 50 minimization steps, ring bond lengths (*d*_*ij*_) and bond angles (θ_*ijk*_) are compared to reference values from the initial structure. If any ring exhibits deviations exceeding user constraints from interval bond length and angles, all atoms in the affected ring are immediately frozen, preventing further deformation while allowing other molecular regions to continue optimizing.

##### Step 3: Final Unconstrained Minimization

Following compression, a brief final minimization (200 steps using the same 4-step iterative protocol whose parameterization follows step 1) removes residual strain without biasing forces, distance restraints, or position constraints. This allows the structure to relax freely to a local force field energy minimum while maintaining the surface-adapted geometry established in step 2.

## Supplementary Material



## Data Availability

Data to evaluate the claims are available in the main text or in the Supporting Information. MISO code embedded into the SXM viewer is available on https://github.com/Anggara-Group/MISO.

## Notes

Description of peptide and lipid assembly (PDF)
