# Supplementary material for: Automating Model Building for SPM Images of Biomolecules Using MISO
Source: J Chem Inf Model. 2026 Aug 3;66(16):10056–69. doi: 10.1021/acs.jcim.6c01134 (PMC13508773; doi:10.1021/acs.jcim.6c01134)
Supplement: Supplementary file 1 [file ci6c01134_si_001.pdf]

Supporting Information

Automating model building for SPM images of biomolecules using MISO

Claudia L. Gomez-Flores[1]
Kelvin Anggara[1]

[1]Max Planck Institute for Solid State Research, DE-70569 Stuttgart, Germany

Contents

1 Peptide building **S2**

2 Lipid building **S2**

3 Bond creation **S5**

3.1 Glycosidic Bond S5

3.2 Hydroxyl group identification S5

4 Glycan assembly **S5**

5 Rotate **S7**

6 Input files **S7**

1 Peptide building

As with the pyranose monomers, peptide residues are mapped onto STM blob positions. When additional experimental data for functional group positions is available (such as side chain termini or aromatic ring centroids), rotational alignment is performed to improve structural accuracy. For amino acids with aromatic side chains (Phe, Tyr, Trp, His), the functional group position corresponds to the ring centroid rather than a single atom, with ring information extracted from the molecular graph's ring detection algorithm. For a ring $\mathcal{R} = \{v_1, v_2, \ldots, v_m\}$ containing m atoms, the centroid is computed as in Eq.1:

$$\mathbf{c}_{\text{ring}} = \frac{1}{m} \sum_{j=1}^{m} \mathbf{x}_{v_j} \tag{1}$$

Glycosylation sites are identified through graph pattern matching on the peptide molecular structure, with each amino acid type exhibiting characteristic functional group connectivity patterns. For asparagine N-glycosylation, the target nitrogen N_δ is part of a side chain amide group with structure $-CH_2 - C(=O) - NH_2$. The identification algorithm searches for nitrogen atoms that are connected to a carbon bearing a double-bonded oxygen (carbonyl group) and have at least one hydrogen attached. This pattern uniquely identifies the side chain amide nitrogen while excluding the backbone amide, which has different connectivity.

For serine and threonine O-glycosylation, the hydroxyl oxygen O_γ is identified by its characteristic pattern: an oxygen atom connected to exactly two neighbors—one carbon (via single bond) and one hydrogen. This two-neighbor constraint ensures selection of the side chain hydroxyl rather than backbone carbonyl oxygens, which lack hydrogen atoms and have different bonding patterns.

For tyrosine O-glycosylation, the phenolic oxygen O_η is distinguished by its attachment to an aromatic carbon within the phenyl ring, combined with the presence of a hydrogen atom. This aromatic ring membership differentiates the phenolic hydroxyl from other oxygen-containing groups in the structure.

Finally, for tryptophan C-glycosylation, the C2 indole carbon is identified through a two-step process: first locating the aromatic nitrogen within the five-membered indole ring, then identifying the aromatic carbon neighbor of this nitrogen. This systematic approach leverages the unique bicyclic structure of the indole side chain.

These graph-based identification methods ensure unambiguous assignment of glycosylation sites by exploiting the distinct chemical connectivity patterns of each amino acid's functional groups, thereby avoiding incorrect matches to structurally similar but chemically distinct groups elsewhere in the peptide.

2 Lipid building

Lipid chains adopt an extended zigzag (all-trans) conformation in their lowest energy state. The carbon backbone is constructed using tetrahedral geometry

with bond angle $\theta_{\text{tet}} = 109.47$. For a chain extending in direction $\hat{\mathbf{d}}$, each carbon alternates laterally to form the characteristic zigzag pattern. Starting from position $\mathbf{c}_0$, a chain of N carbons is generated iteratively. The tetrahedral deviation angle from the main axis corresponds to $\alpha_{\text{dev}} = \frac{\theta_{\text{tet}}}{2} \approx 54.735$. For carbon i, we define the alternating lateral sign $s_i = (-1)^i$ and compute a perpendicular vector (Eq.2) to the main direction:

$$\hat{\mathbf{p}} = \begin{cases} \frac{\hat{\mathbf{d}} \times [0,0,1]^T}{\|\hat{\mathbf{d}} \times [0,0,1]^T\|} & \text{if } |d_z| < 0.9 \\ \frac{\hat{\mathbf{d}} \times [1,0,0]^T}{\|\hat{\mathbf{d}} \times [1,0,0]^T\|} & \text{otherwise} \end{cases} \tag{2}$$

The position of carbon i is computed via the displacement vector seen in Eq.3:

$$\Delta \mathbf{c}_i = \ell_{C-C} \left[\cos(\alpha_{\text{dev}}) \hat{\mathbf{d}} + s_i \sin(\alpha_{\text{dev}}) \hat{\mathbf{p}} \right] \tag{3}$$

where $\ell_{C-C} = 1.54$ Å is the standard C-C single bond length. The cumulative position follows as $\mathbf{c}_i = \mathbf{c}_0 + \sum_{j=1}^{i} \Delta \mathbf{c}_j$. This construction produces a planar zigzag with alternating $\pm\alpha_{\text{dev}}$ deviations from the main axis, characteristic of all-trans alkyl chains.

The lipid chain attachment point depends on the anchoring mode. For attachment to a sugar carbon C_k (sugar-anchored mode), the anchor position is offset from the carbon position $\mathbf{c}_k$ toward the target to prevent steric clashes with the sugar ring. Given target position $\mathbf{t}_{\text{target}}$ (peptide N or blob center), we compute the direction vector seen in Eq.4:

$$\hat{\mathbf{d}}_{\text{target}} = \frac{\mathbf{t}_{\text{target}} - \mathbf{c}_k}{\|\mathbf{t}_{\text{target}} - \mathbf{c}_k\|} \tag{4}$$

The anchor position with offset distance $\delta_{\text{offset}} = 3.5$ Å is then Eq. (5):

$$\mathbf{a}_{\text{anchor}} = \mathbf{c}_k + \delta_{\text{offset}} \hat{\mathbf{d}}_{\text{target}} \tag{5}$$

This offset orients the lipid headgroup favorably toward the target while maintaining adequate clearance from the pyranose ring. For branching from an existing lipid chain (lipid-anchored mode), the anchor is identified by finding the nearest carbon to a specified connection blob position $\mathbf{b}_{\text{conn}}$ (Eq. (6)):

$$\mathbf{a}_{\text{branch}} =_{\mathbf{c}_j \in \mathcal{C}_{\text{lipid}}} \|\mathbf{c}_j - \mathbf{b}_{\text{conn}}\| \tag{6}$$

where $\mathcal{C}_{\text{lipid}}$ is the set of carbons in existing lipid chains. No offset is applied for branched attachments, using the carbon position directly.

The alkyl backbone extends from the headgroup terminal carbon toward a target position (peptide nitrogen, blob center, or membrane position). Two strategies are employed depending on geometric complexity. For simple geometries, a single-segment chain from starting position $\mathbf{s}$ to target $\mathbf{t}$ is constructed by computing (Eq. (7)):

$$d_{\text{total}} = \|\mathbf{t} - \mathbf{s}\|, \quad \hat{\mathbf{d}} = \frac{\mathbf{t} - \mathbf{s}}{d_{\text{total}}} \tag{7}$$

The required carbon count is estimated as in Eq. (8):

$$N_{\text{carbons}} = \max\left(2, \left\lfloor \frac{d_{\text{total}}}{\ell_{C-C}} + 0.5 \right\rfloor\right) \tag{8}$$

The zigzag chain is then constructed using the iterative method described above. The final carbon position $\mathbf{c}_N$ typically deviates from the target by $\epsilon_{\text{reach}} = \|\mathbf{c}_N - \mathbf{t}\|$. For sugar-anchored chains, $\epsilon_{\text{reach}} \in [0.5, 3.0]$ Å is typical and acceptable for subsequent molecular dynamics relaxation.

For complex geometries requiring path control (such as avoiding steric clashes or following membrane curvature), a waypoint $\mathbf{w}$ is specified and the chain is constructed in two segments. The first segment extends from $\mathbf{s}$ to $\mathbf{w}$ with distance $d_1 = \|\mathbf{w}-\mathbf{s}\|$, direction $\hat{\mathbf{d}}_1 = \frac{\mathbf{w}-\mathbf{s}}{d_1}$, and carbon count $N_1 = \max\left(2, \left\lfloor \frac{d_1}{\ell_{C-C}} + 0.5 \right\rfloor\right)$. The second segment extends from $\mathbf{w}$ to $\mathbf{t}$ with corresponding parameters $d_2 = \|\mathbf{t}-\mathbf{w}\|$, $\hat{\mathbf{d}}_2 = \frac{\mathbf{t}-\mathbf{w}}{d_2}$, and $N_2 = \max\left(2, \left\lfloor \frac{d_2}{\ell_{C-C}} + 0.5 \right\rfloor\right)$. The total chain length is $N_{\text{total}} = N_1 + N_2$, with Segment 2 beginning at the terminal carbon of Segment 1. This approach provides greater control over chain trajectory while maintaining the zigzag conformation within each segment.

An optional inverse kinematics refinement step is available to improve target coordination at the cost of minor conformational distortions; for structures undergoing subsequent molecular dynamics relaxation this step is generally omitted.

For complete atomic structures, hydrogen are added to the carbon backbone using tetrahedral geometry. For an internal carbon $\mathbf{c}_i$ with neighbors $\mathbf{c}_{i-1}$ and $\mathbf{c}_{i+1}$, we define the bisector vector as Eq. (9):

$$\mathbf{b}_i = \frac{\mathbf{c}_{i-1} - \mathbf{c}_i}{\|\mathbf{c}_{i-1} - \mathbf{c}_i\|} - \frac{\mathbf{c}_{i+1} - \mathbf{c}_i}{\|\mathbf{c}_{i+1} - \mathbf{c}_i\|} \tag{9}$$

After normalization $\hat{\mathbf{b}}_i = \mathbf{b}_i/\|\mathbf{b}_i\|$, the two hydrogen positions for methylene (CH_2) groups are placed at $\mathbf{h}_{i,1} = \mathbf{c}_i + \ell_{C-H}\hat{\mathbf{b}}_i$ and $\mathbf{h}_{i,2} = \mathbf{c}_i - \ell_{C-H}\hat{\mathbf{b}}_i$, where $\ell_{C-H} = 1.09$ Å.

For terminal methyl (CH_3) groups, the terminal carbon $\mathbf{c}_N$ with single neighbor $\mathbf{c}_{N-1}$ defines the primary direction $\hat{\mathbf{d}}_{\text{back}} = \frac{\mathbf{c}_{N-1}-\mathbf{c}_N}{\|\mathbf{c}_{N-1}-\mathbf{c}_N\|}$. Perpendicular vectors are computed as $\hat{\mathbf{p}}_1 = \frac{\hat{\mathbf{d}}_{\text{back}} \times \mathbf{v}_\perp}{\|\hat{\mathbf{d}}_{\text{back}} \times \mathbf{v}_\perp\|}$ and $\hat{\mathbf{p}}_2 = \hat{\mathbf{d}}_{\text{back}} \times \hat{\mathbf{p}}_1$. The three hydrogen positions are arranged tetrahedrally with factor $\gamma = 0.942$ as in Eq. (10):

$$\begin{aligned}
\mathbf{h}_{N,1} &= \mathbf{c}_N - \ell_{C-H}\hat{\mathbf{d}}_{\text{back}} \\
\mathbf{h}_{N,2} &= \mathbf{c}_N + \gamma\ell_{C-H}\left[\cos(0)\hat{\mathbf{p}}_1 + \sin(0)\hat{\mathbf{p}}_2\right] \\
\mathbf{h}_{N,3} &= \mathbf{c}_N + \gamma\ell_{C-H}\left[\cos(2\pi/3)\hat{\mathbf{p}}_1 + \sin(2\pi/3)\hat{\mathbf{p}}_2\right]
\end{aligned} \tag{10}$$

This produces the characteristic three-fold symmetry of the methyl group, completing the all-atom representation of the lipid chain.

3 Bond creation

3.1 Glycosidic Bond

Glycosidic bond formation is implemented through a purely geometric algorithm that constructs the bridging oxygen position from first principles. Given the three-dimensional coordinates of the donor anomeric carbon and the acceptor carbon, the bridging oxygen position $\mathbf{O}$ is computed as in Eq. (11):

$$\mathbf{O} = \mathbf{C_1} + d\cos(\theta)\,\hat{\mathbf{u}} \pm d\sin(\theta)\,\hat{\mathbf{n}} \tag{11}$$

where $\hat{\mathbf{u}} = (\mathbf{C_2} - \mathbf{C_1})\,/\,|\mathbf{C_2} - \mathbf{C_1}|$ is the unit vector along the C1–C2 axis, $d = 1.43$ Å is the target C–O bond length, θ is derived from the law of cosines applied to the C1–O–C2 triangle with a fixed bond angle of 117.0°, and $\hat{\mathbf{n}}$ is a unit vector perpendicular to $\hat{\mathbf{u}}$ constructed via cross product. Anomeric configuration is encoded by the sign of the perpendicular term: the β configuration retains $+\hat{\mathbf{n}}$, placing the bridging oxygen on the default face of the donor ring, while the α configuration inverts to $-\hat{\mathbf{n}}$, displacing the oxygen to the opposite face. This avoids explicit torsional sampling while correctly encoding stereochemistry.

3.2 Hydroxyl group identification

Hydroxyl group identification at a target carbon is performed from the molecular bond graph. During molecule loading, an RDKit connectivity analysis precomputes a mapping (`oh_map`) that associates each ring carbon with its exocyclic hydroxyl oxygen and that oxygen's hydrogen. At bond-formation time, the flagged oxygen and its hydrogen, when present, are removed directly at the target carbon.

4 Glycan assembly

The final assembly of the glycan structure consists on the combination of individually positioned monomers into a single RDKit object. Monomers are first collected with their 3D coordinates and concatenated using `Chem.CombineMols`, after which glycosidic bonds and functional group decorations are applied in successive passes. Each pass may remove atoms — hydroxyl groups, amines, or hydrogens — from the combined molecule, which requires continuous maintenance of global atom indices. Since RDKit reassigns all atom indices upon each call to `RemoveAtom`, a bookkeeping function tracks how many atoms with indices smaller than a given target have been removed, applying the cumulative offset (Eq. (12)):

$$i_{\text{adj}} = i_{\text{orig}} - |\{r \in R \mid r < i_{\text{orig}}\}| \tag{12}$$

where R is the set of removed global indices and i_{orig} is the pre-removal index. All atom removals within a given pass are therefore collected and sorted in

reverse order before execution, ensuring that each deletion does not invalidate the indices of atoms yet to be removed in the same pass.

For glycopeptide structures, covalent bonds between sugar anomeric carbons and peptide nitrogen or oxygen atoms introduce rotatable linkages whose torsional state is not constrained by the geometry construction algorithm. Trans configuration is enforced explicitly after all bonds are created and a single sanitization pass is performed. The sanitization is intentionally deferred to this point: applying it after each bond addition risks intermediate valence violations on partially bonded atoms, which would raise exceptions and corrupt the molecule state. Once the topology is complete, trans geometry is imposed by identifying the four-atom dihedral sequence $[a_1, C_{\text{sugar}}, N/O_{\text{peptide}}, a_4]$, where a_1 and a_4 are the first available neighbors of the terminal atoms, and setting Eq. (13):

$$\phi(a_1, C_{\text{sugar}}, X_{\text{peptide}}, a_4) = \pi \tag{13}$$

via `rdMolTransforms.SetDihedralRad`. This hard assignment bypasses torsional sampling and directly encodes the energetically preferred trans conformation observed in experimentally resolved glycopeptide structures. The trans geometry established here is preserved through the subsequent force-field optimization. The initial constrained phase freezes the glycosidic atoms as a rigid anchor, after which the restraint alone holds the linkage trans while permitting the bond to relax to its equilibrium length.

Following assembly, systematic positional drift is corrected by realigning sugar ring centers of mass and peptide Cα positions to their experimentally derived coordinates. Since each monomer object retains its identity and reference COM from the building stage, ring matching requires no proximity search — each pyranose ring is directly associated with its source monomer and retranslated accordingly. Throughout the monomer building pipeline, quaternion-based rigid body transformations were deliberately chosen precisely because they preserve internal geometry exactly, accumulate no numerical error under composition, and guarantee that each monomer enters the assembly with correct Cartesian coordinates. However, once monomers are transferred into the RDKit object, quaternion operations can no longer be applied. RDKit's conformer manipulation is restricted to direct Cartesian coordinate assignment, and its atom removal and bond insertion routines operate without bonded-geometry awareness or rigid body constraint propagation. Each atom deletion and bond addition therefore modifies the conformer independently, causing small but systematic positional deviations that accumulate across assembly passes and cannot be avoided within the RDKit framework. The COM restoration and Cα realignment steps are consequently a necessary correction to recover the geometric accuracy that the quaternion pipeline guaranteed during building.

For peptide residues, each Cα atom is translated to its target position and the side-chain functional group is subsequently reoriented by a 2D rotation in the xy-plane around the Cα, aligning the current functional group vector to its experimental counterpart. This restoration step is essential for maintain-

ing agreement with the experimental reference geometry from which monomer coordinates were originally derived, and ensures that the exported structure is suitable for direct comparison with crystallographic or cryo-EM data without requiring post-hoc energy minimization to recover correct ring and backbone positions.

Prior to optimization, explicit hydrogens are added to the assembled structure via OpenBabel's `AddHydrogens` routine. The protonated structure is then converted back to RDKit via an SDF round-trip and sanitized. This conversion can fail for structures with non-standard valence states arising from the assembly stage, in which case the OpenBabel molecule is returned directly and flagged for manual inspection before entering the subsequent custom force-field optimization stage.

5 Rotate

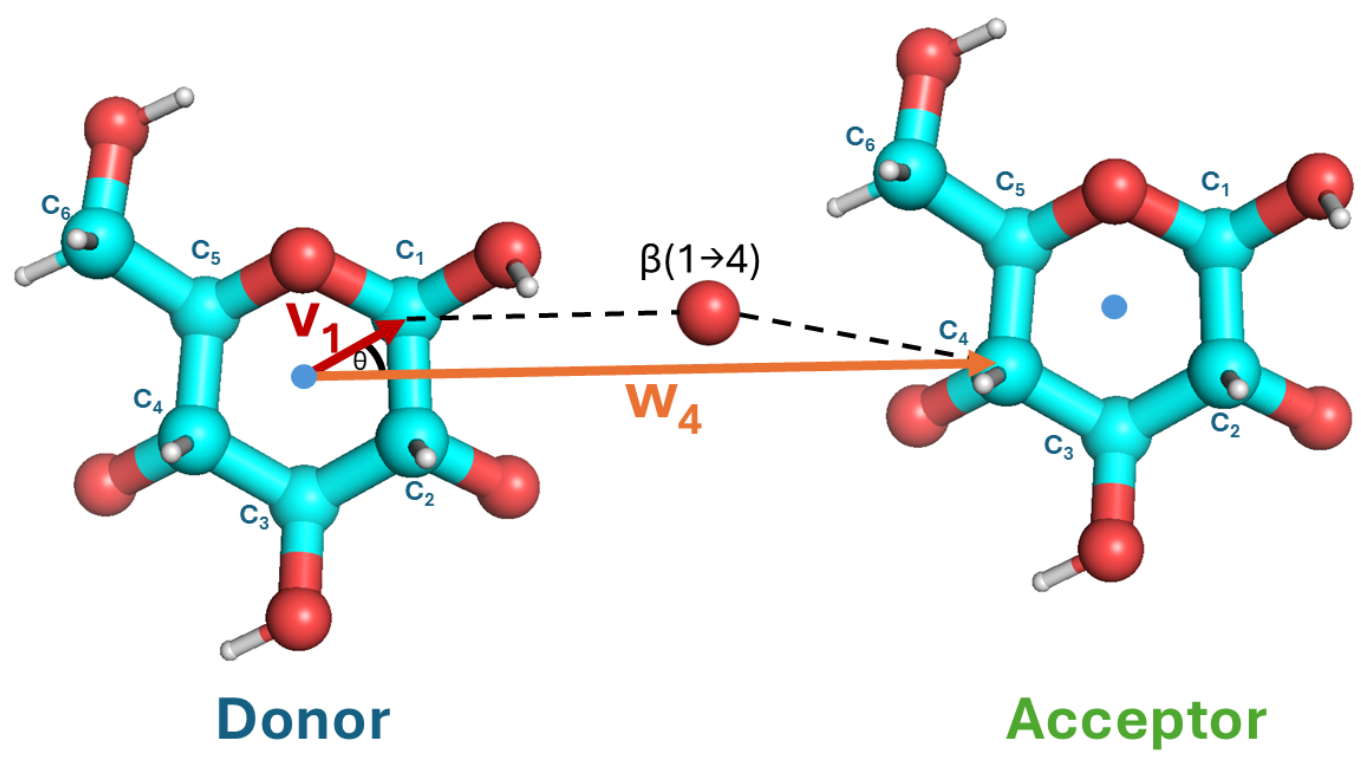

S1: Definition of dC_i, v_i, and w_i vectors used in MISO-ROTATE.

As an example of how QUEST aligns two glucose subunits, several vectors are defined. dC_i is defined as a vector from the center-of-mass toward the i-th C-atom, whereas v_i is the normalized dC_i vector (as exemplified by the red arrow in the figure, denoted as v_i - which is the normalized dC_1 vector). w_i is defined as a vector from the center of mass of the donor glucose subunit towards the C-atom in the acceptor glucose subunit (as exemplified by the orange arrow in the figure, denoted as w_4 - which is the w-vector from donor center-of-mass to the C_4 of the acceptor glucose).

6 Input files

For generating the GM3 ganglioside we used the following input file:

```yaml
sxm_file: '210605_Cu100_024.sxm'
circle_input_path: '210605_Cu100_024_coordinates.csv'
stm_grid_path: '210605_Cu100_024.npz'

sugars:
  Gal: 'C([C@@H]1[C@@H]([C@@H]([C@H](C(O1)O)O)O)O)O'
  SIA: 'CC(=O)N[C@@H]1[C@H](CC(O[C@H]1[C@@H]([C@@H](CO)O)O)(C(=O)O)O)O'
  Glc: 'C([C@@H]1[C@H]([C@@H]([C@H](C(O1)O)O)O)O)O'

experimental_positions:
  SIA: 7
  Gal: 6
  Glc: 5

Lipids:
 - direction: [2]
   midpoint: [3]
   linkage: 'amide'
   connection_blob: [4]
   connection_anchor: 'lipid'
 - direction: [0]
   midpoint: [1]
   connection: ['Glc', 'C1']
   linkage: 'ether'
   connection_anchor: 'sugar'

conformer_parameters:
  num_conformers: 10
  max_keep: 1

conformer_selection:
  strategy: 'lowest_energy'

root_mol: 'Gal'

direction:
  - ['Glc', 'C1',[4]]
  - ['SIA', 'C6',[8]]
  - ['SIA', 'C4',[9]]
  - ['Gal', 'C6',[10]]
  - ['Glc', 'C6',[11]]
  - ['Glc', 'C4','Gal', 'C1']
  - ['Gal', 'C3','SIA', 'C1']

glycosidic_bonds:
  - ['Glc', 'C4','Gal', 'C1', 'beta', 'Gal-Glc beta(1-4)']
```

```
  - ['Gal', 'C3','SIA', 'C1', 'alpha', 'SIA-Gal alpha(2-3)']
```

For generating the NGA2F N-glycopeptide on the KVANKT peptide we used the following input file:

```
sxm_file: 'fa.sxm'
circle_input_path: 'fa_coordinates.csv'
stm_grid_path: 'fa.npz'

sugars:
  GlcNAc: 'CC(=O)N[C@@H]1[C@H]([C@@H]([C@H](OC1O)CO)O)O'
  Fuc: 'C[C@H]1[C@H]([C@H]([C@@H](C(O1)O)O)O)O'
  Man: 'C([C@@H]1[C@H]([C@@H]([C@@H](C(O1)O)O)O)O)O'

experimental_positions:
  GlcNAc: [0, 5,7,10]
  Fuc: 9
  Man: [2,3,4]

conformer_parameters:
  num_conformers: 20
  max_keep: 3

conformer_selection:
  strategy: 'lowest_energy'

root_mol: 'Fuc'

direction:
  - ['GlcNAc_0', 'C1', 'Man_2', 'C2']
  - ['GlcNAc_0', 'C2', [1]]
  - ['Man_2', 'C1', 'Man_3', 'C6']
  - ['Man_3', 'C3', 'Man_4', 'C1']
  - ['Man_4', 'C2', 'GlcNAc_5', 'C1']
  - ['GlcNAc_5', 'C2', [6]]
  - ['Man_3', 'C1', 'GlcNAc_7', 'C4']
  - ['GlcNAc_7', 'C2', [8]]
  - ['GlcNAc_7', 'C1', 'GlcNAc_10', 'C4']
  - ['GlcNAc_10', 'C3', 'Fuc', 'C1']
  - ['GlcNAc_10', 'C2', [11]]
  - ['GlcNAc_10', 'C1', [12]]



peptide:
```

```
  sequence: 'K-V-A-N-K-T'
  residues:
    - aa: 'Lys'
      ca_position: 15
      functional_position: 16
    - aa: 'Val'
      ca_position: 13
      functional_position: 14
    - aa: 'Ala'
      ca_position: 12
      functional_position: null
    - aa: 'Asn'
      ca_position: 21
      functional_position: 22
    - aa: 'Lys'
      ca_position: 17
      functional_position: 18
    - aa: 'Thr'
      ca_position: 19
      functional_position: 20
peptide_bonds:
  - sugar_mol: 'GlcNAc_10'
    sugar_carbon: 'C1'
    aa_type: 'Asn'
    residue_index: 3
    glycosylation_type: 'N-glycosidic'
    anomeric_config: 'beta'
    use_spacer: false

glycosidic_bonds:
- ['GlcNAc_0', 'C1', 'Man_2', 'C2', 'beta', 'GlcNAc_0-Man_2 beta(1-2)']
- ['Man_2', 'C1', 'Man_3', 'C6', 'alpha', 'Man_2-Man_3 alpha(1-6)']
- ['Man_3', 'C3', 'Man_4', 'C1', 'alpha', 'Man_3-Man_4 alpha(3-1)']
- ['Man_4', 'C2', 'GlcNAc_5', 'C1', 'beta', 'Man_4-GlcNAc_5 beta(2-1)']
- ['Man_3', 'C1', 'GlcNAc_7', 'C4', 'beta', 'Man_3-GlcNAc_7 beta(1-4)']
- ['GlcNAc_7', 'C1', 'GlcNAc_10', 'C4', 'beta', 'GlcNAc_7-GlcNAc_10 beta(1-4)']
- ['GlcNAc_10', 'C3', 'Fuc', 'C1', 'alpha', 'GlcNAc_10-Fuc_11 alpha(3-1)']
```

For generating the glycosaminoglycan (GAG) oligosaccharide we used the following input file:

```
sxm_file: 'hs1.sxm'
circle_input_path: 'hs1_coordinates.csv'
stm_grid_path: 'hs1.npz'
```

```
sugars:
  GlcNAc: 'CC(=O)N[C@@H]1[C@H]([C@@H]([C@H](OC1O)CO)O)O'
  GlcA: '[C@@H]1([C@@H]([C@H](OC([C@@H]1O)O)C(=O)O)O)O'
experimental_positions:
  GlcNAc: [0,2,4,6]
  GlcA: [1,3,5,7]

conformer_parameters:
  num_conformers: 20
  max_keep: 3

conformer_selection:
  strategy: 'lowest_energy'

root_mol: 'GlcA_7'

direction:
  - ['GlcNAc_0', 'C1', 'GlcA_1', 'C4']
  - ['GlcA_1', 'C1', 'GlcNAc_2', 'C4']
  - ['GlcNAc_2', 'C1', 'GlcA_3', 'C4']
  - ['GlcA_3', 'C1', 'GlcNAc_4', 'C4']
  - ['GlcNAc_4', 'C1', 'GlcA_5', 'C4']
  - ['GlcA_5', 'C1', 'GlcNAc_6', 'C4']
  - ['GlcNAc_6', 'C1', 'GlcA_7', 'C4']
  - ['GlcNAc_0', 'C2', [9]]
  - ['GlcNAc_2', 'C2', [10]]
  - ['GlcNAc_4', 'C2', [11]]
  - ['GlcNAc_6', 'C2', [12]]
  - ['GlcNAc_6', 'C5', [13]]

glycosidic_bonds:
- ['GlcNAc_0', 'C1', 'GlcA_1', 'C4', 'beta', 'GlcNAc-GlcA beta(1-4)']
- ['GlcA_1', 'C1', 'GlcNAc_2', 'C4', 'beta', 'GlcA-GlcNAc beta(1-4)']
- ['GlcNAc_2', 'C1', 'GlcA_3', 'C4', 'beta', 'GlcNAc-GlcA beta(1-4)']
- ['GlcA_3', 'C1', 'GlcNAc_4', 'C4', 'beta', 'GlcA-GlcNAc beta(1-4)']
- ['GlcNAc_4', 'C1', 'GlcA_5', 'C4', 'beta', 'GlcNAc-GlcA beta(1-4)']
- ['GlcA_5', 'C1', 'GlcNAc_6', 'C4', 'beta', 'GlcA-GlcNAc beta(1-4)']
- ['GlcNAc_6', 'C1', 'GlcA_7', 'C4', 'beta', 'GlcNAc-GlcA beta(1-4)']
```

For generating the Glc-Glc-AsnPheAla glycopeptide we used the following input file:

sxm_file: 'glycopeptide.sxm'
circle_input_path: 'glycopeptide_coordinates.csv'
stm_grid_path: 'glycopeptide.npz'

sugars:
  Glc: 'C([C@@H]1[C@H]([C@@H]([C@H](C(O1)O)O)O)O)O'

experimental_positions:
  Glc: [0, 1]

conformer_parameters:
  num_conformers: 20
  max_keep: 3

conformer_selection:
  strategy: 'lowest_energy'

root_mol: 'Glc_0'

direction:
  - ['Glc_0', 'C1', 'Glc_1', 'C4']
  - ['Glc_1', 'C6', [7]]

peptide:
  sequence: 'A-F-N'
  residues:
    - aa: 'Asn'
      ca_position: 2
      functional_position: 3
    - aa: 'Phe'
      ca_position: 4
      functional_position: 5
    - aa: 'Ala'
      ca_position: 6
      functional_position: null

peptide_bonds:
  - sugar_mol: 'Glc_1'
    sugar_carbon: 'C1'
    aa_type: 'Asn'
    residue_index: 2
    glycosylation_type: 'N-glycosidic'
    anomeric_config: 'beta'
    use_spacer: false

glycosidic_bonds:

- ['Glc_0', 'C1', 'Glc_1', 'C4', 'beta', 'Glc-Glc beta(1-4)']
